# Real-Time Polymerase Chain Reaction Systems for Detection and Differentiation of Unclassified Viruses of the *Phenuiviridae* Family

**DOI:** 10.3390/mps8010020

**Published:** 2025-02-18

**Authors:** Alena V. Dereventsova, Alexander S. Klimentov, Ivan S. Kholodilov, Oxana A. Belova, Alexander M. Butenko, Galina G. Karganova

**Affiliations:** 1 Laboratory of Biochemistry, Chumakov Federal Scientific Center for Research and Development of Immune-and-Biological Products of RAS (Institute of Poliomyelitis), 108819 Moscow, Russia; 2 Laboratory of Biology of Arboviruses, Chumakov Federal Scientific Center for Research and Development of Immune-and-Biological Products of RAS (Institute of Poliomyelitis), 108819 Moscow, Russia; 3 D.I. Ivanovsky Institute of Virology Division of N.F. Gamaleya National Research Center of Epidemiology and Microbiology of the Ministry of Health of the Russian Federation, 1230986 Moscow, Russia; 4 Institute for Translational Medicine and Biotechnology, Sechenov University, 119991 Moscow, Russia

**Keywords:** *Phenuiviridae*, RT-PCR, tick-borne viruses, *Uukuvirus*, *Ixovirus*, *Phlebovirus*, Stavropol virus, Pedaselga virus, Kizhi virus, Gomselga virus

## Abstract

The family *Phenuiviridae*, part of the order *Hareavirales*, includes arboviruses and arthropod-associated viruses, with sandflies, mosquitoes, and ticks as primary vectors. Historically, only sandfly/mosquito-borne phenuiviruses were associated with human diseases, but the emergence of severe fever with thrombocytopenia syndrome (SFTS) has highlighted the potential of tick-borne phenuiviruses as human pathogens. Recent discoveries of new arthropod-associated viruses, some of which remain unclassified, underscore the need for sensitive detection and differentiation methods, particularly in regions where these viruses may co-circulate. This study aimed to develop real-time PCR test systems for identifying and differentiating unclassified viruses within the *Phenuiviridae* family. In this study, tick suspensions containing phenuiviruses, previously obtained during the screening of ticks from various regions of Russia using pan-phenuivirus primers, were used. Specific primers and probes were designed to differentiate five *Phenuiviridae* viruses of genera *Uukuvirus*, *Ixovirus*, *Phlebovirus* and one unclassified phenuivirus, and their analytical sensitivity and specificity were evaluated. These PCR-based tools provide a robust method for detecting and classifying uncharacterized phenuiviruses, contributing to improved surveillance and understanding their potential epidemiological and epizootological impacts.

## 1. Introduction

The family *Phenuiviridae* is recognized as the largest and most diverse family within the order *Hareavirales*, formerly *Bunyavirales*, which was renamed two years ago, comprising 23 genera and 159 species capable of infecting animals, plants, and fungi, along with a large number of unclassified viruses. Many members are arboviruses, which are associated with arthropods such as ticks, mosquitoes, and sandflies [1]. Viruses of the *Phenuiviridae* family are widely distributed across Asia, Europe, Africa, and North America [2,3,4,5]. Some pose risks to human and animal health as potential sources of emerging arthropod-borne infectious diseases, while the pathogenicity of others remains unidentified. Recently, there has been growing interest in the full genome sequences of tick-borne phenuiviruses.

Phenuivirus virions are spherical, approximately 100 nanometers in diameter, and their genome consists of three segments of single-stranded RNA with negative-sense and ambisense polarity. The large (L) segment encodes an RNA-dependent RNA polymerase that also functions as an endonuclease and participates in mRNA cap snatching. The medium (M) segment encodes precursors of surface glycoproteins Gn and Gc, which mediate virion binding to target cells, cytosolic entry, and virion assembly. In some viruses, the M segment also encodes a nonstructural protein, NSm, known to interact with the outer mitochondrial membrane and inhibit apoptosis [6,7]. The small (S) segment encodes both the nucleocapsid protein N, which encapsulates genomic RNA, and the nonstructural protein NSs, which modulates interferon production in host cells and regulates viral virulence [8].

Recent advancements in high-throughput sequencing and PCR systems have led to discovery of the new tick-associated viruses that exhibit genetic similarity to known pathogenic phenuiviruses [9,10]. However, it remains unclear whether these newly identified viruses can cause novel infectious diseases in humans and animals. Given the widespread distribution and genetic diversity of phenuiviruses, it is plausible that some tick-borne human diseases may be caused by underexplored members of this family. This highlights the need for further research to understand the nature of tick-associated phenuiviruses, their ecological roles, and their potential to emerge as new infectious disease agents.

From a medical perspective, the most significant interest lies in viruses from the genera *Phlebovirus* and *Bandavirus*. In addition to these viruses, which are known to have the potential to infect humans and animals, there are several genera, such as *Uukuvirus* and *Ixovirus*, whose members have been detected in arthropods but their arboviral potential and pathogenicity remain undefined.

The largest genus within the *Phenuiviridae* family is *Phlebovirus*, which includes 67 species, such as highly pathological *Phlebovirus riftense* and sandfly fever viruses like *Phlebovirus toscanaense*. Viruses of this genus infect mammals, including humans, and are transmitted by mosquitoes and sandflies and are distributed all over the world. Some viruses of the genus *Phlebovirus* are associated with fatal febrile illnesses and encephalitis in humans. Cases of congenital malformations and abortions in livestock have also been reported [11,12]. *Phlebovirus mukawaense* is a tick-derived virus that exhibits greater genetic similarity to sandfly/mosquito-borne phleboviruses than to other tick-borne phenuiviruses. Neutralizing antibodies were detected in mammals and even though it was shown that it has a potential to infect human cell lines, its pathogenicity still remains unexplored [10,13].

Another significant genus in the *Phenuiviridae* family is *Bandavirus*. Members of this genus infect humans as well as other vertebrates. Human cases caused by the tick-borne *Bandavirus dabieense* have been reported in China, South Korea, Japan, and Vietnam, with both humans and domestic or wild animals being susceptible [4,14]. Clinical symptoms include high fever, thrombocytopenia, leukopenia, gastrointestinal disorders, hemorrhagic manifestations, neurological symptoms associated with encephalitis, and multiple organ dysfunctions [2,4]. Asymptomatic infections have also been documented. The tick-borne *Bandavirus heartlandense* was first identified in 2009 in two patients in the United States, and specific antibodies have since been detected in wild animals in the eastern and central regions of the U.S. [5,15]. The tick-borne *Bandavirus bhanjanagarense* has been detected in Asia, Africa, and Europe [3]. Although it rarely causes illness, it is regularly identified, including in the Russian Federation [16]. The *Bandavirus kismaayoense* has been minimally described in the literature so far, and its pathogenic potential is not yet fully understood. Furthermore, within these groups, there are both pathogenic and nonpathogenic viruses. Studying and comparing pathogenic and nonpathogenic viruses within the same group may provide insights into the mechanisms underlying their virulence.

Antibodies against viruses of the genus *Uukuvirus* have been found in the sera of birds and other mammals, including humans, though these viruses have not been linked to any diseases [17].

The RNA of ixoviruses has been detected in ticks. The genome of ixoviruses consist of two segments encoding L and N proteins, the presence of an M segment has not been confirmed, and vertebrate hosts remain unidentified [18]. Likewise, recent studies of some members of the genera *Phlebovirus* and *Ixovirus* could not identify the M segment [19]. This absence is unusual for *Hareavirales*, as the M segment is a critical structural and functional component of this order. Additionally, some cases have been reported where the M segment is present but does not encode the nonstructural protein NSm, which plays a key role in infecting *Aedes aegypti* mosquitoes, for example [20]. Several theories have been proposed to explain these anomalies. One suggests that these viruses may employ mechanisms for cell attachment and entry that do not involve glycoproteins [21]. Another posits that the L and S segments may encode an episomal form of the virus, utilizing alternative transmission mechanisms [19]. This genomic diversity within the *Phenuiviridae* family underscores the need for further molecular studies to uncover novel pathways of viral entry and transmission.

Numerous endogenous viral elements related to phenuiviruses have been documented, particularly those associated with polymerase or nucleocapsid proteins, as well as some previously linked to glycoproteins [22]. Sequence analyses from multiple regions have not revealed clear geographic specificity [23,24], which aligns with the detection of similar viruses in widely separated areas and emphasizes the need for comprehensive epidemiological surveillance. It remains unclear what advantages the integration of genome segments provides to ticks. This warrants further investigation and must be considered when developing diagnostic tools.

Thus, recent discoveries of phenuiviruses provide a unique opportunity to study arthropod virus evolution, molecular features, and replication mechanisms during host alternation, as well as their virulence in vertebrates. These viruses exhibit high genetic diversity, and accumulating genomic data—including full genome sequences and individual segments, particularly L and S—enables the study of their co-evolution with hosts [25,26]. Investigating virus distribution across different tick species may reveal arboviral potential and the risks of novel virus variants arising from reassortment. Association with a single arthropod species suggests transovarial and trans-stadial transmission as primary model that reduces the likelihood of the emergence of a new virus variant that will be pathogenic for vertebrates. However, it is hypothesized that phenuiviruses also undergo horizontal transmission, enabling genetic recombination with other viruses, parasites, or host RNA within the same organism [27,28]. This process could facilitate adaptation to diverse hosts and the emergence of novel viruses with unique traits, complicating phylogenetic analyses [29].

A significant diversity and wide distribution of phenuiviruses across the Russian Federation have been demonstrated [25,30]. These viruses are prevalent in various regions, including the Astrakhan Region, Belgorod Region, Kaliningrad Region, Kaluga Region, Republic of Karelia, Krasnodar Region, Moscow Region, Ryazan’ Region, Saratov Region, Stavropol Territory, Republic of Tatarstan, Republic of Tuva, Ulyanovsk Region, Voronezh Region, and Chelyabinsk Region. From 2005 to 2018, extensive data collection and analysis of ticks during their peak activity periods were conducted in these regions. PCR analysis of collected ticks identified 12 distinct groups of phenuiviruses based on nucleotide sequence analysis [25,30,31]. In some cases, the RNA of multiple viruses was detected in a single tick species in the same area, suggesting co-infection with multiple viruses within one tick species [25].

Particular interest lies in the genera *Phlebovirus*, *Uukuvirus,* and *Ixovirus*, which include Gomselga (*Phlebovirus*), Stavropol (*Uukuvirus*), Pedaselga (*Ixovirus*), and Kizhi (*Ixovirus*) viruses, as well as the unclassified Andropov virus, since these viruses are widely distributed in Russia and belong to genera whose representatives have the potential to be or are arboviruses.

To detect viruses, the molecular PCR method is used, targeting the most conserved regions of viral genomes, followed by sequencing to determine the specific virus species. Real-time polymerase chain reaction (RT-PCR) is characterized by its high sensitivity, specificity, quantitative capabilities, efficiency, and speed. This method can detect low levels of viral nucleic acids, making it particularly useful for the early detection and monitoring of viral infections with low viral loads. By designing specific primers and probes targeting unique genomic regions, RT-PCR enables precise identification and differentiation of various viral strains/isolates [32].

Additionally, the multiplexing capability of RT-PCR offers several advantages, such as the simultaneous detection and differentiation of multiple strains/isolates or virus types in a single reaction. This reduces sample processing errors, minimizes contamination risk, and allows easy scalability of analyses, which is particularly important when processing large number of samples. Currently, no real-time test system for the detection and differentiation of unclassified phenuiviruses has been published.

The aim of this study is to develop RT-PCR test systems for the detection and differentiation of unclassified *Phenuiviridae* viruses in tick suspensions and biological material.

## 2. Materials and Methods

### 2.1. Virus-Containing Ticks

The study utilized virus-containing tick suspensions from the collection of the Laboratory of Biology of Arboviruses at the Chumakov Federal Scientific Center for Research and Development of Immune-and-Biological Products, RAS (Institute of Poliomyelitis) [25,30]. Their general characteristics are presented in Table 1.

Tick suspensions of *Ixodes persulcatus*, *Dermacentor reticulatus*, *Hyalomma scupense*, and *Hyalomma marginatum* collected in the same regions were used as negative controls. Tick-borne encephalitis virus (TBEV; NCBI accession number of sequence: OQ673267) and *Uukuvirus uukuniemiense* (NCBI accession number of sequence: NC005214), also used as negative controls, were described in previous studies [30,31].

The *Bandavirus bhanjanagarense* (NCBI accession number of sequence: KC521440) and *Bandavirus kismaayoense* (NCBI accession number of sequence: PQ740907) strains used as negative controls in this study were kindly provided from the collection of the Laboratory of Arbovirus Biology and Detection at the N.F. Gamaleya National Research Center for Epidemiology and Microbiology [31].

The poliovirus strain Sabin from the Institute of Poliomyelitis’ collection was used as the internal control for this study. The previously designed oligonucleotides with a fluorescent probe were used [33].

### 2.2. RNA Isolation, PCR, and Sequencing

RNA extraction was performed from 125 µL of the tick suspension or virus-containing culture fluid using TRI Reagent (Sigma-Aldrich, St. Louis, MO, USA) according to the manufacturer’s protocol [25].

A fixed amount (5.5 log RNA copies per sample) of the attenuated poliovirus type I Sabin strain was added as an internal control. Reverse transcription was performed with Random Primer (Evrogen, Moscow, Russia) and M-MLV reverse transcriptase (Evrogen, Moscow, Russia) according to the manufacturer’s protocol.

cDNA amplification was performed using PCR with gene-specific primers (forward: 5′-GGCTACTTCAARAAYAARGANGA-3′; reverse: 5′-CARCAYGGIGGICTGAGAGAG-3′) [31]. Each PCR reaction included 2 µL of cDNA, 5 µL of 10× PCR buffer (Thermo Fisher Scientific, Waltham, MA, USA), 0.16 µM of each primer in a volume of 1.5 µL, 2.5 μL of a 2.5 mM dNTP mix, and 0.5 µL of DreamTaq polymerase (Thermo Fisher Scientific, Waltham, MA, USA) in 50 µL. The amplification conditions were as follows: 20 s at 95 °C, 30 s at 50 °C, and 60 s at 72 °C, repeated for 40 cycles.

PCR products were visualized on a 1% agarose gel. Gel purification was performed using the QIAquick Gel Extraction Kit (Qiagen, Düsseldorf, Germany). Sequencing was carried out using an Applied Biosystems 3130 Genetic Analyzer. The obtained sequences were analyzed using Lasergene^®^SeqMan Pro Software Version 7.0.0 (DNASTAR Inc., Madison, WI, USA).

### 2.3. Phylogenetic Analysis

The RNA sequences of some representatives of the family *Phenuiviridae* and the isolates described in this article were used in phylogenetic analysis. The nucleotide sequences of fragments of the L segment (nt positions 2297 to 2806) were aligned using ClustalW. Phylogenetic analysis was conducted using the maximum likelihood method and the Tamura–Nei model [34] in MEGA X with 1000 bootstrap replications [35].

### 2.4. Primers Selection for Phenuiviruses Differentiating Test Systems

Primers were designed based on available virus sequences [31].

The selection of primers and probes was carried out using the online program Real-time PCR (TaqMan) Primer and Probes Design Tool (GenScript, Oxford, UK). The oligonucleotides targeted the L segment of each virus. The constructed specific primers and probes were synthesized by Evrogen (Moscow, Russia). The compositions of the constructed primers and probes, as well as the selected fluorescent markers, are listed in Table 2.

### 2.5. Test System Validation

A commercial standardized R-412 kit (Syntol, Moscow, Russia) was used to perform real-time PCR with fluorescence in situ hybridization detection. Amplification of the samples was carried out using the QuantStudio5 amplifier (Thermo Fisher Scientific, Waltham, MA, USA). The obtained amplification data of analytical sensitivity of the real-time PCR systems were analyzed using Bio-Rad CFX Manager v.3.1 (Bio-Rad, Hercules, CA, USA).

A total of 2.5 μL of 10× PCR buffer B, 2.5 μL of a 2.5 mM dNTP mix, 2.5 μL of 25 mM MgCl_2_, 10 pmol of oligonucleotide primers, 10 pmol of the fluorescent probe, and 1 unit of SynTaq DNA polymerase in a volume of 0.2 μL were added to 2 μL of cDNA, and the final volume was adjusted to 25 μL with deionized water (all the reagents were supplied by Syntol, Moscow, Russia).

The same reagents were used for duplex. Two µL of cDNA from each virus being studied and 10 pmol of oligonucleotide primers and fluorescent probes for each virus included in the analysis were added to the mix. The mixture was then diluted with deionized water to a total volume of 25 µL.

The following conditions were set for the amplification reaction: 2 min at 50 °C, 10 min at 95 °C, 20 s at 95 °C, and 40 s at 60 °C. The reaction cycle was repeated 40 times.

Analytical sensitivity was evaluated using tenfold serial dilutions of positive control samples for each virus.

## 3. Results

### 3.1. Phylogenetic Relationships Within the Phenuiviridae Family

**Figure 1:**
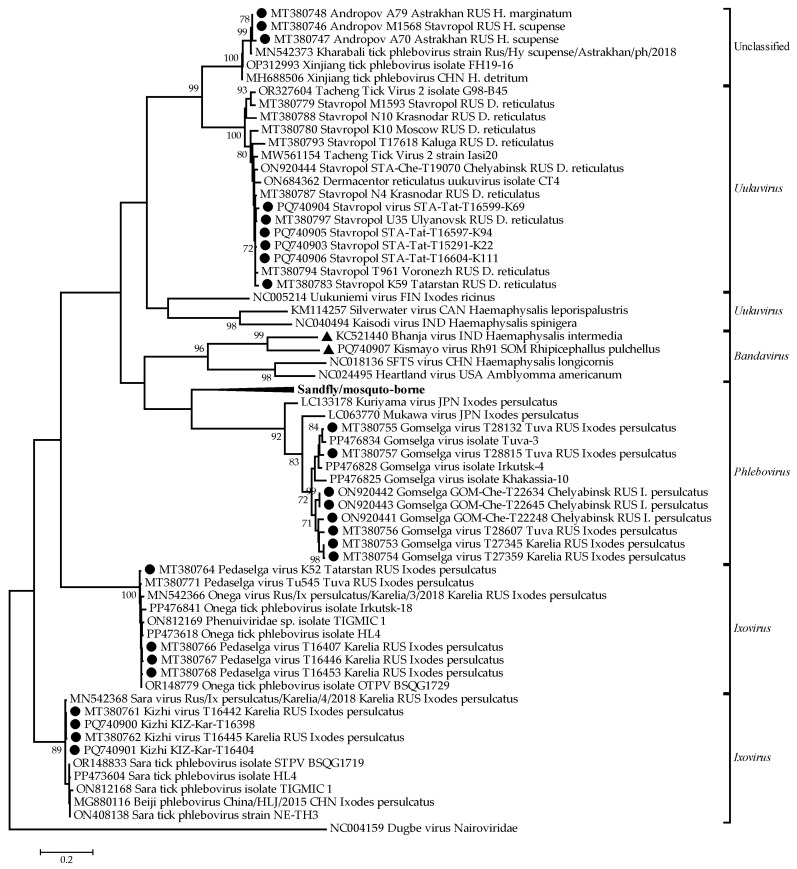
Phylogenetic analysis of the family *Phenuiviridae*. Phylogenetic trees were constructed using fragments of the L segment (nt positions 2297 to 2806) in MEGA X with the maximum likelihood method (1000 bootstrap replications). Black circles—isolates of the phenuiviruses described in this study. Black triangles—strains of the phenuiviruses used in this study as a control.

In addition to the previously described isolates [25,30,31], we isolated and sequenced four isolates of the Stavropol virus (PQ740903–PQ740906) and three isolates of the Kizhi virus (PQ740900–PQ740902). The RNA sequences of some representatives of the family *Phenuiviridae* and the isolates described in this article were used in phylogenetic analysis (Figure 1).

The phylogenetic tree revealed distinct clustering patterns among the analyzed phenuivirus strains, supporting their classification within the *Phenuiviridae* family (Figure 1). The unclassified phenuiviruses formed a separate cluster from the established genera, suggesting the possibility of novel classifications. Additionally, the *Uukuvirus* and *Bandavirus* clades contained reference sequences alongside closely related strains described in this study, indicating a strong evolutionary relationship.

A distinct monophyletic branch was observed for the sandfly/mosquito-borne phenuiviruses, reflecting their unique evolutionary lineage. Within the *Phlebovirus* group, multiple Gomselga virus isolates described in this study clustered together, demonstrating a notable regional presence. Similarly, the *Ixovirus* cluster exhibited significant genetic diversity, forming a closely related group, potentially indicating evolutionary divergence within this lineage.

### 3.2. Primer Selection for Phenuiviruses Differentiating Test Systems

Nucleotide sequences of viruses from the collection of the Laboratory of Biology of Arboviruses at the Chumakov Federal Scientific Center for Research and Development of Immune-and-Biological Products were analyzed at the initial stage. The most conserved regions specific to each virus species were selected, and specific nucleotide primers and probes were designed. The probes contained a fluorophore at the 5′ end and a fluorescence quencher at the 3′ end.

Virus-specific RT-PCR test systems were developed for detecting the Stavropol, Andropov, Pedaselga, Kizhi, and Gomselga phenuiviruses. Since the Pedaselga, Kizhi, and Gomselga viruses are associated with *I. persulcatus* ticks, duplex real-time PCR test systems were designed to detect multiple viruses in a single assay. Primers and probes for all target regions were optimized to ensure specific and efficient amplification of the target sequences.

Nine virus-specific test systems were tested on virus-containing arthropod suspensions:

- Three for detecting the Stavropol virus;
- Two, for the Pedaselga virus;
- Two, for the Andropov virus;
- Two, for the Kizhi virus;
- Three, for the Gomselga virus.

All primers and probes used in the study were tested for specificity. To determine the most sensitive test system for each virus, analyses were conducted under identical experimental conditions using cDNA from several isolates of the same virus. The reactions were performed under the same conditions.

The results of the PCR reactions were analyzed by comparing the threshold cycle (Ct) values for the test systems as shown in Table S1. For further work, the sets of primers and probes showing the lowest Ct values were selected. These were as follows:

- St-rt4 for Stavropol;
- And-rt2 for Andropov;
- Pd-rt1 for Pedaselga;
- Kz-rt2 for Kizhi;
- Gom-rt3 for Gomselga.

### 3.3. Evaluation of Specificity for Uniplex Test Systems

cDNA from the following arboviruses was used for specificity assessment: tick-borne encephalitis virus, *Bandavirus bhanjanagarense*, *Uukuvirus uukuniemiense*, *Bandavirus kismaayoense*, as well as cDNA from the poliovirus. The developed sets of primers and probes for all the tested viruses successfully and specifically amplified the cDNA of the target virus without amplifying any of the negative control viruses. The test systems were supplemented with a poliovirus internal control to prevent false-negative results. The threshold cycle values for these test systems are presented in Table 3. Exponential growth in fluorescence channels was observed during the experiments, as shown in Figure S2, corresponding to each of the identified pathogens.

### 3.4. Sensitivity Evaluation of the Test Systems

Samples with the highest viral load were used for sensitivity analysis, and ten-fold dilutions were prepared (Table 4). The standard curve is constructed by plotting the dilution factor against the Ct value obtained during amplification of each dilution. The coefficients of determination (R^2^), the slopes of the curves, and the amplification efficiency are shown in Table 5. The equations of the linear regression line and the linear dependence between the dilution degree and the Ct are shown in Figures S3–S7. In undiluted samples, the Ct values were as follows:

- Stavropol virus: 21.3;
- Andropov virus: 23.4;
- Pedaselga virus: 23.3;
- Kizhi virus—unable to detect due to overload;
- Gomselga virus—unable to detect due to overload.

The limit of detection was defined by deionized water used as a negative control.

The results showed that all the viruses were successfully detected up to a dilution of 10^−3^. However, at a 10^−4^ dilution, the Ct values for the Stavropol, Andropov, and Pedaselga viruses approached the threshold cycle values of the negative control.

The amplification curves for the ten-fold dilution series of each virus showed a clear linear relationship between the dilution factor and the Ct values (Figures S3–S7). The R^2^ values ranged from 0.98 to 0.99, indicating strong linearity and reliable quantification across the dilution series (Table 5). The slopes of the standard curves ranged from −3.13 to −3.57, and amplification efficiency varied between 90.43% and 108.61% (Table 5).

The sensitivity of the developed PCR test systems is sufficient to detect viral particles, even in highly diluted samples. The Ct values provide critical information about viral concentration and help evaluate the efficiency and detection limits of the PCR test systems. Exponential growth in fluorescence channels is shown in Figures S8–S12.

### 3.5. Evaluation of Duplex Test Systems

Various fluorescent markers with unique wavelengths were used to visualize each sample in the multiplex reaction. The fluorescent signal indicating the amplification of the specific genome fragment of the Pedaselga virus was detected in the FAM channel, while the Kizhi and Gomselga viruses were identified in the ROX and Cy5 channels, respectively.

The same sets of primers and probes used in the uniplex analysis were employed for the duplex reaction. Both sets capable of detecting the studied viruses were placed in the reaction mixture, with cDNA of the studied viruses used as a template. During RT-PCR, exponential growth of fluorescent signals was observed in the FAM, ROX, and Cy5 channels, corresponding to each identified pathogen.

Duplex test systems showed successful detection and differentiation of the analyzed viruses (Table 6 and Table 7). This system was less sensitive compared to the uniplex RT-PCR. However, in some cases, the differences in the threshold cycle of the two systems were insignificant. Despite its lower sensitivity, the duplex systems have sufficient sensitivity to detect viral RNA and differentiate viruses.

Based on the data obtained, one can infer that the developed RT-PCR system will enhance the speed and efficiency of molecular identification of unclassified phenuiviruses in biological material.

## 4. Discussion

The wide variety of phenuiviruses, their extensive prevalence, and their unexplored nature necessitates the development of new methods for their detection and identification to enable effective epidemiological surveillance and timely public health interventions. Traditional methods of phenuivirus detection often require subsequent sequencing to accurately determine the virus species, making the diagnostic process more time consuming and costly.

In this study, an RT-PCR system was developed for the detection and differentiation of five uncharacterized viruses of the *Phenuiviridae* family that were found in Russia and phylogenetically associated with different genera: Stavropol (*Uukuvirus*), Pedaselga (*Ixovirus*), Kizhi (*Ixovirus*), Gomselga (*Phlebovirus*), and the unclassified phenuivirus Andropov. Our studies show that the presented phenuiviruses are associated with a specific tick species. Stavropol virus is associated with *Dermacentor reticulatus* [25], which is confirmed by other studies where Tacheng Tick virus 2 [36,37] and Dermacentor reticulatus uukuvirus [38], which form one phylogenetic group with Stavropol virus, were isolated from the *Dermacentor reticulatus* tick. Viruses from three monophyletic groups are associated with the *Ixodes persulcatus* tick. The first group contains the Pedaselga virus [25] and the Onega tick phlebovirus [39]; the second group contains the Kizhi virus [25], Sara tick phlebovirus, and Beiji phlebovirus; and the third group contains Gomselga virus, all isolates of which were obtained from *Ixodes persulcatus* collected in regions far from each other [25,30]. Additionally, the Andropov virus is linked to *Hyalomma scupense*; however, some isolates from the Astrakhan Region are associated with both *Hyalomma scupense* and *Hyalomma marginatum* [25].

The developed test systems demonstrated high specificity in amplifying the DNA of target viruses while avoiding amplification between each other and other arboviruses, which is particularly important in cases requiring accurate diagnosis or identification. A clear linear relationship between the dilution factor and the Ct values confirm the reliability of the test systems. The R^2^ values demonstrate strong linearity and reliable quantification throughout the dilution series. The slopes of the standard curves were close to the slope of an ideal standard curve [40]. The range of the amplification efficiencies was within the optimal range (90–110%) [40], ensuring accurate and efficient amplification. Notably, the Pedaselga and Gomselga test systems showed slightly higher efficiencies (108.61% and 105.58%, respectively), suggesting potential minor variations in amplification conditions that may warrant further optimization. Overall, high specificity and sensitivity make the developed test systems versatile tools suitable for primary screening as well as confirmatory diagnostics. This minimizes diagnostic errors and improves overall result accuracy, enhancing their clinical significance. Further optimization could be considered for assays with efficiencies slightly above 100% to ensure consistent performance.

The use of a duplex RT-PCR test system for simultaneous virus detection further improved the efficiency of molecular identification. Optimization of primers and probes for each target virus, along with the use of distinct fluorescent markers for visualization, ensured specific and efficient amplification of viral genomes. The inclusion of positive and negative control samples in multiplex reactions confirmed the reliability of the results.

It is worth noting that while the duplex RT-PCR method enabled the detection and differentiation of target viruses within a single analysis, it showed a slightly lower sensitivity compared to uniplex reactions. Further optimization of this system is required. Additionally, developing multiplex test systems capable of simultaneous detection of a greater number of phenuiviruses represents a promising direction.

This study makes an important contribution to the development of phenuivirus diagnostic methods. The developed test systems enable more effective epidemiological surveillance and timely measures for the prevention and control of phenuivirus infections. Further research and validation of these test systems on various biological matrices and under field conditions will be necessary to fully realize their application in public health and epidemiological studies aimed at understanding these pathogens.

## Figures and Tables

**Table 1:** Isolates of viruses used for testing oligonucleotides and probes.

Isolate	Collection Site and Date	Tick Species	Number of Pools	Number of Ticks	NCBI Number
Stavropol	Republic of Tatarstan, 2012	*Dermacentor* *reticulatus*	5	12	MT380783, PQ740903–PQ740906
	Ulyanovsk Region, 2014	*Dermacentor* *reticulatus*	1	5	MT380797
Andropov	Stavropol Krai, 2011–2012	*Hyalomma scupense*	1	22	MT380746
	Astrakhan Region, 2015	*Hyalomma scupense* *Hyalomma* *marginatum*	2	20	MT380747, MT380748
Pedaselga	Republic of Karelia, 2012	*Ixodes persulcatus*	3	3	MT380766–MT380768
	Republic of Tatarstan, 2012	*Ixodes persulcatus*	1	1	MT380764
Kizhi	Republic of Karelia, 2012	*Ixodes persulcatus*	5	5	MT380761, MT380762, PQ740900–PQ740902
Gomselga	Chelyabinsk Region, 2015	*Ixodes persulcatus*	3	17	ON920441– ON920443
	Republic of Karelia, 2018	*Ixodes persulcatus*	2	10	MT380753, MT380754
	Republic of Tuva, 2018	*Ixodes persulcatus*	3	13	MT380755–MT380757

**Table 2:** The sequences of primers and probes selected for the detection of unclassified phenuiviruses.

Virus	Location	Oligonucleotide	Sequence
Stavropol	L segment	St-rtF1	5′-GGCTATGGYGAYCCHCCIYT-3′
		St-rtR1	5′-TGAGHGTRGAYACTCTATCC-3′
		St-rtPr1	(6-FAM)-TCTGCAACYTGKCCCTRATCATGYT-(BHQ-1)
		St-rtF2	5′-CAACYTGKCCCTRATCATGY-3′
		St-rtR2	5′-TYTCCACCACCTTCYTCCKR-3′
		St-rtPr2	(6-FAM)-RCCYTCWAACACGCAYGTGCAYCA-(BHQ-1)
		St-rtF4	5′-TAGAGTRTCYACICTMAAGG-3′
		St-rtR4	5′-CTGYYTCCAGGCATTGCTTY-3′
		St-rtPr4	(6-FAM)-RCCYTCWAACACGCAYGTGCAYCA-(BHQ-1)
Andropov	L segment	And-rtF1	5′-GAGTGATGGCTYGAAACAGT-3′
		And-rtR1	5′-TCGCTTCTCAATGGTCTCCA-3′
		And-rtPr1	(6-FAM)-ACACCCATGTGCACCACGTTYTG-(BHQ-1)
		And-rtF2	5′-ACTCAACTCCAGCACTCATCA-3′
		And-rtR2	5′-TGGGTTCTCATGTCACCCAAW-3′
		And-rtPr2	(ROX)-ACTGGAGGACCARCACCCTGG-(BHQ-2)
Pedaselga	L segment	Pd-rtF1	5′-TCTCTGGGTGGCTACTCTCT-3′
		Pd-rtR1	5′-GGTRGSACYTCTTRGGATCG-3′
		Pd-rtPr1	(6-FAM)-AGCCTTTAGCGTTCCGAGCTGC-(BHQ-1)
		Pd-rtF2	5′-CRCTAAAGGCWTCCTCAACC-3′
		Pd-rtR2	5′-TCATGCACATGAGTGTGCTC-3′
		Pd-rtRt2	(6-FAM)-ACGATCCYAAGAWGTSCYACCACAGG-(BHQ-1)
Kizhi	L segment	Kz-rtF1	5′-ATCAGCCACGTACGATCAGA-3′
		Kz-rtR1	5′-AGTCCCTCCAAACACTCCTG-3′
		Kz-rtPr1	(ROX)-TCACAGAGCAACCCACATYCATGAGCT-(BHQ-2)
		Kz-rtF2	5′-TACTGTGCTGACTCTGCCAy-3′
		Kz-rtR2	5′-GTGGCTGATGCTTTCAGTGT-3′
		Kz-rtPr2	(ROX)-TGCCATTGGCAACTACGACCTCG-(BHQ-2)
Gomselga	L segment	Gm-rtF2	5′-CCACCCTHAAGGCCACATCR
		Gm-rtR2	5′-AGYCTCTTTCCTGCYCTYGT
		Gm-rtPr2	(Cy5)-TGTRCACTCYGARAACAAGGGAMGA-(BHQ-2)
		Gm-rtF3	5′-TRCACTCYGARAACAAGGGA-3′
		Gm-rtR3	5′-GCTCTCCTGRAATGCCTCTA-3′
		Gm-rtPr3	(Cy5)-AGTTGCmCCrYTGACrAGrGCAGG-(BHQ-2)
		Gm-rtF5	5′-RGGTAyCTAGGGAArCAGGA-3′
		Gm-rtR5	5′-CTCAAGrGTGAGGTGGCAGA-3′
		Gm-rtPr5	(Cy5)-YCCTsTrAAGCCYACYATGCATGAGT-(BHQ-2)

**Table 3:** Threshold cycle values for cDNA analysis of tested viruses.

Virus	Sample	Test System St-rt4 *	Test System And-rt2 *	Test System Pd-rt1 *	Test System Kz-rt2 *	Test System Gom-rt3 *
Stavropol	PQ740903	22.8 ± 1.3	-	-	-	-
	MT380783	18.4 ± 0.4	-	-	-	-
	PQ740904	23.7 ± 0.9	-	-	-	-
	PQ740905	16.2 ± 0.5	-	-	-	-
	PQ740906	20.9 ± 0.5	-	-	-	-
	MT380797	22.4 ± 0.5	-	-	-	-
Andropov	MT380746	-	24.1 ± 0.8	-	-	-
	MT380747	-	22.7 ± 0.9	-	-	-
	MT380748	-	24.9 ± 0.6	-	-	-
Pedaselga	MT380766	-	-	28.1 ± 0.8	-	-
	MT380767	-	-	23.4 ± 1.1	-	-
	MT380768	-	-	26.1 ± 1.2	-	-
	MT380764	-	-	21.8 ± 0.9	-	-
Kizhi	MT380761	-	-	-	4.9 ± 1.2	-
	MT380762	-	-	-	6.2 ± 0.6	-
	PQ740902	-	-	-	12.7 ± 1.2	-
	PQ740900	-	-	-	18.8 ± 0.9	-
	PQ740901	-	-	-	21.6 ± 1.3	-
Gomselga	ON920441	-	-	-	-	21.8 ± 0.6
	ON920442	-	-	-	-	16.5 ± 1.8
	ON920443	-	-	-	-	17.8 ± 0.7
	MT380753	-	-	-	-	28.4 ± 0.4
	MT380754	-	-	-	-	6.8 ± 1.1
	MT380755	-	-	-	-	20.2 ± 1.3
	MT380756	-	-	-	-	22.0 ± 0.8
	MT380757	-	-	-	-	19.7 ± 1.7
*Tick-borne* *encephalitis virus*	OQ673267	-	-	-	-	-
*Bandavirus bhanjanagarense*	KC521440	-	-	-	-	-
*Bandavirus kismaayoense*	PQ740907	-	-	-	-	-
*Uukuvirus uukuniemiense*	NC005214	-	-	-	-	-
Unclassified tick suspensions						
	*Dermacentor reticulatus*	-	-	-	-	-
	*Hyalomma scupense*	-	-	-	-	-
	*Hyalomma marginatum*	-	-	-	-	-
	*Ixodes persulcatus*	-	-	-	-	-

* Average Ct of three RT-PCR runs. “-” stands for no amplification.

**Table 4:** Analytical sensitivity of the real-time PCR systems.

Dilution of the Control Sample	Ct Stavropol	Ct Andropov	Ct Pedaselga	Ct Kizhi	Ct Gomselga
no dilution	21.3	23.4	23.3	-	-
10^−1^	29.3	30.3	27.5	-	4.8
10^−2^	31.9	32.5	30.6	4.2	9.1
10^−3^	35.9	35.9	33.7	11.6	12.6
10^−4^	38.9	39.3	36.2	13.4	14.5
10^−5^	N/A	N/A	37.3	17.1	17.6
10^−6^	N/A	N/A	N/A	20.9	21.6
10^−7^	-	-	-	24.2	24.3
10^−8^	-	-	-	27.2	29.1
10^−9^	-	-	-	31.8	30.1
10^−10^	-	-	-	34.8	N/A
10^−11^	-	-	-	N/A	N/A
N/C	N/A	N/A	N/A	N/A	N/A

“N/C” stands for negative control; “N/A” stands for no amplification.

**Table 5:** Standard curve parameters of the real-time PCR systems.

Parameters of the Real-Time PCR	Test System St-rt4	Test System And-rt2	Test System Pd-rt1	Test System Kz-rt2	Test System Gom-rt3
R^2^	0.99	0.99	0.98	0.99	0.99
Slope	−3.54	−3.48	−3.13	−3.57	−3.19
Efficiency *	91.64	93.80	108.61	90.43	105.58

* The amplification efficiency presented in percentage.

**Table 6:** The threshold value via FAM/ROX channels with simultaneous cDNA analysis of Pedaselga and Kizhi viruses.

Sample	Ct *, FAM Channel	Ct *, ROX Channel	Ct *, Uniplex
Pedaselga (MT380766)	30.1 ± 0.7	-	28.1 ± 0.8
Kizhi (PQ740900)	-	19.7 ± 0.6	18.8 ± 0.9
Pedaselga (MT380767)	25.7 ± 0.8	-	23.4 ± 1.1
Kizhi (MT380762)	-	7.5 ± 0.5	6.2 ± 0.6
Pedaselga (MT380768)	27.5 ± 0.9	-	26.1 ± 1.2
Kizhi (PQ740902)	-	20.5 ± 0.6	12.7 ± 1.2

* Average Ct of three RT-PCR runs.

**Table 7:** The threshold value via ROX/Cy5 channels with simultaneous cDNA analysis of Kizhi and Gomselga viruses.

Sample	Ct *, ROX Channel	Ct *, Cy5 Channel	Ct *, Uniplex
Kizhi (PQ740900)	20.4 ± 0.9	-	18.8 ± 0.9
Gomselga (ON920441)	-	25.9 ± 0.7	21.8 ± 0.9
Kizhi (MT380762)	8.5 ± 0.9	-	6.2 ± 0.6
Gomselga (ON920442)	-	18.9 ± 0.7	16.5 ± 1.8
Kizhi (PQ740902)	14.7 ± 0.8	-	12.7 ± 1.2
Gomselga (ON920443)	-	18.5 ± 0.9	17.8 ± 0.8

* Average Ct of three RT-PCR runs.

## Data Availability

The data presented in the study are available in the manuscript.

## Supplementary Materials

The following supporting information can be downloaded at: https://www.mdpi.com/article/10.3390/mps8010020/s1, Figure S1: Fragments of L segment alignment. Table S1: Threshold cycle values for developed test systems. Figure S2: Fluorescence curves on the optical channels of the QuantStudio5 amplifier. Figures S3–S7: The linear dependence between the dilution degree and the threshold cycle. Figures S8–S12: Amplification curve for the samples of diluted virus.
