# Supplementary material for: Real-Time Polymerase Chain Reaction Systems for Detection and Differentiation of Unclassified Viruses of the Phenuiviridae Family
Source: Methods Protoc. 2025 Feb 18;8(1):20. doi: 10.3390/mps8010020 (PMC11857896; doi:10.3390/mps8010020)
Supplement: Supplementary file 1 [file mps-08-00020-s001.zip › mps-3398075-supplementary.pdf]

**A.**

```
                        St-rtF1                  St-rtPr1                      St-rtR1
                        2425                2444 2492                     2516 2592                2611
                        ....|....|....|....|/////....|....|....|....|....|/////....|....|....|....|
Stavropol_set_1_oligs   GGCTATGGYGAYCCHCCIYT-----TCTGCAACYTGKCCCTRATCATGYT-----GGATAGAGTRTCYACDCTCA
Stavropol_U35           GGCTATGGTGACCCTCCTTT-----TCTGCAACCTGGCCCTAATCATGCT-----GGATAGAGTGTCCACGCTCA
Stavropol_U31           GGCTATGGTGACCCTCCTTT-----TCTGCAACCTGGCCCTGATCATGCT-----GGATAGAGTGTCCACACTCA
Stavropol_U10           GGCTATGGTGACCCTCCTTT-----TCTGCAACCTGGCCCTAATCATGCT-----GGATAGAGTGTCCACGCTCA
Stavropol_U9            GGCTATGGCGACCCTCCTTT-----TCTGCAACCTGGCCCTAATCATGCT-----GGATAGAGTGTCCACGCTCA
Stavropol_U7            GGCTATGGTGACCCTCCTTT-----TCTGCAACCTGGCCCTAATCATGCT-----GGATAGAGTGTCCACGCTCA
Stavropol_U6            GGCTATGGTGACCCTCCTTT-----TCTGCAACCTSGCYCTAATCATGCT-----GGATAGAGTGTCCACGCTCA
Stavropol_N96           GGCTATGGTGACCCTCCTTT-----TCTGCAACCTGGCCCTAATCATGCT-----GGATAGAGTGTCCACGCTCA
Stavropol_N95           GGCTATGGTGACCCTCCTTT-----TCTGCAACCTGGCCCTAATCATGCT-----GGATAGAGTGTCCACGCTCA
Stavropol_N93           GGCTATGGTGACCCTCCTTT-----TCTGCAACCTGGCCCTAATCATGCT-----GGATAGAGTGTCCACGCTCA
Stavropol_N63           GGCTATGGTGACCCACCACT-----TCTGCAACCTGGCCCTAATCATGCT-----GGATAGAGTGTCCACGCTCA
Stavropol_N48           -----TGGTGACCCTCCTTT-----TCTGCAACCTGGCCCTAATCATGTT-----GGATAGAGTGTCCACGCTCA
Stavropol_N46           ---------GACCCACCACT-----TCTGCAACCTGGCCCTAATCATGCT-----GGATAGAGTGTCCACGCTCA
Stavropol_N23           GGCTATGGTGACCCACCACT-----TCTGCAACCTGGCCCTAATCATGTT-----GGATAGAGTGTCCACGCTCA
Stavropol_N12           gGCTATGGTGACCCACCACT-----TCTGCAACCTGGCCCTAATCATGTT-----GGATAGAGTGTCCACGCTCA
Stavropol_N11           GGCTATGGTGACCCACCACT-----TCTGCAACCTGGCCCTAATCATGTT-----GGATAGAGTGTCCACGCTCA
Stavropol_N10           GGCTATGGTGACCCACCACT-----TCTGCAACTTGGCCCTAATCATGTT-----GGATAGAGTGTCCACGCTCA
Stavropol_N4            GGCTATGGTGACCCTCCTTT-----TCTGCAACCTGGCCCTAATCATGCT-----GGATAGAGTGTCCACGCTCA
Stavropol_N3            -----TGGTGACCCTCCTTT-----TCTGCAACCTGGCCCTAATCATGCT-----GGATAGAGTGTCCACGCTCA
Stavropol_N2            GGCTATGGTGACCCACCACT-----TCTGCAACCTGGCCCTAATCATGTT-----GGATAGAGTGTCCACGCTCA
Stavropol_T944          GGCTATGGTGACCCTCCCTT-----TCTGCAACCTGGCCCTGATCATGCT-----GGATAGAGTGTCCACTCTCA
Stavropol_T961          GGCTATGGTGACCCTCCCTT-----TCTGCAACCTGGCCCTGATCATGCT-----GGATAGAGTGTCCACTCTCA
Stavropol_Che325        GGCTATGGTGACCCTCCTTT-----TCTGCAACCTGGCCCTAATCATGCT-----GGATAGAGTGTCCACGCTCA
Stavropol_Che324        GGCTATGGTGACCCTCCTTT-----TCTGCAACCTGGCCCTAATCATGCT-----GGATAGAGTGTCCACGCTCA
Stavropol_Che321        GGCTATGGTGACCCTCCTTT-----TCTGCAACCTGGCCCTAATCATGCT-----GGATAGAGTGTCCACGCTCA
Stavropol_Che320        GGCTATGGTGACCCTCCTTT-----TCTGCAACCTGGCCCTAATCATGCT-----GGATAGAGTGTCCACGCTCA
Stavropol_1590          GGCTATGGTGACCCACCACT-----TCTGCAACCTGGCCCTAATCATGCT-----GGATAGAGTGTCTACGCTCA
Stavropol_1593          GGCTATGGTGACCCACCACT-----TCTGCAACCTGGCCCTAATCATGCT-----GGATAGAGTGTCCACGCTCA
Stavropol_TB1040        GGCTATGGTGACCCACCACT-----TCTGCAACCTGGCCCTAATCATGTT-----GGATAGAGTGTCCACGCTCA
Stavropol_K103          GGCTATGGTGACCCTCCTTT-----TCTGCAACCTGGCCCTAATCATGCT-----GGATAGAGTGTCCACGCTCA
Stavropol_K108          GGCTATGGCGACCCCCCCTT-------------------------------------
Stavropol_k60           GGCTATGGTGACCCTCCTTT-----TCTGCAACCTGGCCCTGATCATGCT-----GGATAGAGTGTCCACKCTCA
Stavropol_k61           GGCTATGGTGACCCCCCTTT-----TCTGCAACCTGGCCCTAATCATGCT-----GGATAGAGTGTCCACGCTCA
Stavropol_k62           GGCTATGGTGACCCTCCTTT-----TCTGCAACCTGTCCCTAATCATGCT-----GGATAGAGTATCCACGCTCA
Stavropol_k63           GGCTATGGTGACCCTCCTTT-----TCTGCAACCTGGCCCTAATCATGCT-----GGATAGAGTGTCCACGCTCA
Stavropol_k65           GGCTATGGTGACCCTCCTTT-----TCTGCAACCTGGCCCTGATCATGCT-----GGATAGAGTGTCCACTCTCA
Stavropol_k66           GGCTATGGTGACCCTCCTTT-----TCTGCAACCTGGCCCTAATCATGCT-----GGATAGAGTGTCCACGCTCA
Stavropol_k69           GGCTATGGTGACCCTCCTTT-----TCTGCAACCTGTCCCTAATCATGCT-----GGATAGAGTATCCACGCTCA
Stavropol_k70           GGCTATGGTGACCCTCCTTT-----TCTGCAACCTGGCCCTAATCATGCT-----GGATAGAGTGTCCACGCTCA
Stavropol_k73           GGCTATGGTGACCCTCCTTT-----TCTGCAACCTGGCCCTAATCATGCT-----GGATAGAGTGTCCACGCTCA
```

```
Stavropol_k74          GGCTATGGTGACCCTCCTTT-----TCTGCAACCTGGCCCTAATCATGCT-----GGATAGAGTGTCCACGCTCA
Stavropol_k75          GGCTATGGTGACCCTCCTCT-----TCTGCAACCTGGCCCTAATCATGCT-----GGATAGAGTGTCCACGCTCA
Stavropol_K59          GGCTATGGTGACCCTCCTTT-----TCTGCAACCTGGCCCTAATCATGCT-----GGATAGAGTGTCCACGCTCA
Stavropol_k58          GGCTATGGCGACCCTCCTTT-----TCTGCAACCTGGCCCTAATCATGCT-----GGATAGAGTGTCCACGCTCA
Stavropol_k28          GGCTATGGTGACCCTCCTTT-----TCTGCAACCTGGCCCTAATCATGCT-----GGATAGAGTGTCCACGCTCA
Stavropol_k22          GGCTATGGYGACCCTCCTTT-----TCTGCAAYCTGGCCCTAATCATGCT-----GGATAGAGTGTCCACGCTCA
Stavropol_K17          GGCTATGGCGACCCTCCTTT-----TCTGCAACCTGGCCCTAATCATGCT-----GGATAGAGTGTCCACGCTCA
Stavropol_k14          GGCTATGGYGACCCTCCTTT-----TCTGCAACCTGGCCCTAATCATGCT-----GGATAGAGTGTCCACGCTCA
Stavropol_k13          GGCTATGGTGACCCTCCTTT-----TCTGCAACCTGGCCCTAATCATGCT-----GGATAGAGTGTCCACGCTSA
Stavropol_k11          GGCTATGGTGACCCCCCTTT-----TCTGCAACCTGGCCCTAATCATGCT-----GGATAGAGTGTCCACGCTCA
Stavropol_k10          GGCTATGGTGATCCTCCGCT-----TCTGCAACCTGGCCCTGATCATGCT-----GGATAGAGTGTCTACGCTAA

                       St-rtF2                 St-rtPr2                     St-rtR2
                       2496               2515 2603                   2625  2670               2689
                       ....|....|....|....|/////....|....|....|....|...////....|....|....|....|
Stavropol_set_2_oligs  CAACYTGKCCCTRATCATGY-----CYACICTMAAGGCRTCYAGTGCY-----YMGGARGAAGGTGGTGGARA
Stavropol_N96          CAACCTGGCCCTAATCATGC-----CCACGCTCAAGGCATCTAGTGCC-----TCGGAAGAAGGTGGTGGAGA
Stavropol_N95          CAACCTGGCCCTAATCATGC-----CCACGCTCAAGGCATCTAGTGCC-----TCGGAAGAAGGTGGTGGAGA
Stavropol_N93          CAACCTGGCCCTAATCATGC-----CCACGCTCAAGGCATCTAGTGCC-----TCGGAAGAAGGTGGTGGAGA
Stavropol_N63          CAACCTGGCCCTAATCATGC-----CCACGCTCAAGGCGTCTAGTGCC-----TAGGAAGAAGGTGGTGGAAA
Stavropol_N48          CAACCTGGCCCTAATCATGT-----CCACGCTCAAGGCATCTAGTGCC-----TCGGAAGAAGGTGGTGGAGA
Stavropol_N46          CAACCTGGCCCTAATCATGC-----CCACGCTCAAGGCGTctAGtGCC-----TAGGAAGAAGGTGGTGGAAA
Stavropol_N23          CAACCTGGCCCTAATCATGT-----CCACGCTCAAGGCATCTAGTGCT-----TAGGAAGAAGGTGGTGGAAA
Stavropol_N12          CAACCTGGCCCTAATCATGT-----CCACGCTCAAGGCATCTAGTGCT-----TAGGAAGAAGGTGGTGGAAA
Stavropol_N11          CAACCTGGCCCTAATCATGT-----CCACGCTCAAGGCATCTAGTGCT-----TAGGAAGAAGGTGGTGGAAA
Stavropol_N10          CAACTTGGCCCTAATCATGT-----CCACGCTCAAGGCATCTAGTGCT-----TAGGAAGAAGGTGGTGGAAA
Stavropol_N4           CAACCTGGCCCTAATCATGC-----CCACGCTCAAGGCATCTAGTGCC-----TCGGAAGAAGGTGGTGGAAA
Stavropol_N3           CAACCTGGCCCTAATCATGC-----CCACGCTCAAGGCATCTAGTGCC-----TCGGAAGAAGGTGGTGGAAA
Stavropol_N2           CAACCTGGCCCTAATCATGT-----CCACGCTCAAGGCATCTAGTGCT-----TAGGAAGAAGGTGGTGGAAA
Stavropol_U35          CAACCTGGCCCTAATCATGC-----CCACGCTCAAGGCATCTAGTGCC-----TCGGAAGAAGGTGGTGGAGA
Stavropol_U31          CAACCTGGCCCTGATCATGC-----CCACACTCAAGGCATCTAGTGCC-----TCGGAAGAAGGTGGTGGAAA
Stavropol_U10          CAACCTGGCCCTAATCATGC-----CCACGCTCAAGGCATCTAGTGCC-----TCGGAAGAAGGTGGTGGAAA
Stavropol_U9           CAACCTGGCCCTAATCATGC-----CCACGCTCAAGGCATCTAGTGCC-----TCGGAAGAAGGTGGTGGAAA
Stavropol_U7           CAACCTGGCCCTAATCATGC-----CCACGCTCAAGGCATCTAGTGCC-----TCGGAAGAAGGTGGTGGAGA
Stavropol_U6           CAACCTSGCYCTAATCATGC-----CCACGCTCAAGGCATCTAGTGCC-----TMGGAAGAAGGTGGTGGARA
Stavropol_T961         CAACCTGGCCCTGATCATGC-----CCACTCTCAAGGCATCTAGTGCC-----TCGGAAGAAGGTGGTGGAAA
Stavropol_T944         CAACCTGGCCCTGATCATGC-----CCACTCTCAAGGCATCTAGTGCC-----TCGGAAGAAGGTGGTGGAAA
Stavropol_Che325       CAACCTGGCCCTAATCATGC-----CCACGCTCAAGGCATCTAGTGCC-----TCGGAAGAAGGTGGTGGAGA
Stavropol_Che321       CAACCTGGCCCTAATCATGC-----CCACGCTCAAGGCATCTAGTGCC-----TCGGAAGAAGGTGGTGGAAA
Stavropol_Che320       CAACCTGGCCCTAATCATGC-----CCACGCTCAAGGCATCTAGTGCC-----TCGGAAGAAGGTGGTGGAAA
Stavropol_Che324       CAACCTGGCCCTAATCATGC-----CCACGCTCAAGGCATCTAGTGCC-----TCGGAAGAAGGTGGTGGAGA
Stavropol_TB1040       CAACCTGGCCCTAATCATGT-----CCACGCTCAAGGCATCTAGTGCT-----TAGGAAGAAGGTGGTGGAAA
Stavropol_K69          CAACCTGTCCCTAATCATGC-----CCACGCTCAAGGCATCTAGTGCC-----TCGGAAGAAGGTGGTGGAAA
Stavropol_k65          CAACCTGGCCCTGATCATGC-----CCACTCTCAAGGCATCTAGTGCC-----TCGGAGGAAGGTGGTGGAAA
```

```
Stavropol_k62              CAACCTGTCCCTAATCATGC-----CCACGCTCAAGGCATCTAGTGCC-----TCGGAAGAAGGTGGTGGAAA
Stavropol_k59              CAACCTGGCCCTAATCATGC-----CCACGCTCAAGGCATCTAGTGCC-----TAGGAAGAAGGTGGTGGAAA
Stavropol_k14              CAACCTGGCCCTAATCATGC-----CCACGCTCAAGGCATCTAGTGCC-----TCGGAAGAAGGTGGTGGAAA
Stavropol_k13              CAACCTGGCCCTAATCATGC-----CCACGCTSAAGGCATCTAGTGCC-----TCGGAAGAAGGTGGTGGAAA
Stavropol_k11              CAACCTGGCCCTAATCATGC-----CCACGCTCAAGGCATCTAGTGCC-----TCGGAAGAAGGTGGTGGAAA
Stavropol_K10              CAACCTGGCCCTGATCATGC-----CTACGCTAAAGGCATCCAGTGCC-----TCGGAAGAAGGTGGTGGAAA
Stavropol_1593             CAACCTGGCCCTAATCATGC-----CCACGCTCAAGGCGTCTAGTGCC-----TAGGAAGAAGGTGGTGGAAA
Stavropol_1590             CAACCTGGCCCTAATCATGC-----CTACGCTCAAGGCGTCTAGTGCC-----TAGGAAGAAGGTGGTGGAAA
Stavropol_K17              CAACCTGGCCCTAATCATGC-----CCACGCTCAAGGCATCTAGTGCC-----TCGGAAGAAGGTGGTGGAAA
Stavropol_k22              CAAYCTGGCCCTAATCATGC-----CCACGCTCAAGGCATCTAGTGCC-----TCGGAAGAAGGTGGTGGARA
Stavropol_k28              CAACCTGGCCCTAATCATGC-----CCACGCTCAAGGCATCTAGTGCC-----TCGGAAGAAGGTGGTGGAGA
Stavropol_k58              CAACCTGGCCCTAATCATGC-----CCACGCTCAAGGCATCTAGTGCC-----TCGGAAGAAGGTGGTGGAAA
Stavropol_k60              CAACCTGGCCCTGATCATGC-----CCACKCTCAAGGCATCTAGTGCC-----TCGGAAGAAGGTGGTGGAGA
Stavropol_k61              CAACCTGGCCCTAATCATGC-----CCACGCTCAAGGCATCTAGTGCC-----TCGGAAGAAGGTGGTGGAAA
Stavropol_k63              CAACCTGGCCCTAATCATGC-----CCACGCTCAAGGCATCTAGTGCC-----TCGGAAGAAGGTGGTGGAAA
Stavropol_k66              CAACCTGGCCCTAATCATGC-----CCACGCTCAAGGCATCTAGTGCC-----TCGGAAGAAGGTGGTGGAGA
Stavropol_k70              CAACCTGGCCCTAATCATGC-----CCACGCTCAAGGCATCTAGTGCC-----TCGGAAGAAGGTGGTGGAGA
Stavropol_k73              CAACCTGGCCCTAATCATGC-----CCACGCTCAAGGCATCTAGTGCC-----CCGGAGGAAGGTGGTGGAGA
Stavropol_k74              CAACCTGGCCCTAATCATGC-----CCACGCTCAAGGCATCTAGTGCC-----TCGGAAGAAGGTGGTGGAAA
Stavropol_k75              CAACCTGGCCCTAATCATGC-----CCACGCTCAAGGCATCTAGTGCC-----TCGGAAGAAGGTGGTGGAAA
Stavropol_K103             CAACCTGGCCCTAATCATGC-----CCACGCTCAAGGCATCTAGTGCC-----TCGGAAGAAGGTGGTGGAGA

                           St-rtF4                  St-rtPr4                      St-rtR4
                           2595                2614 2709                     2732 2739                2758
                           ....|....|....|....|/////....|....|....|....|..../////....|....|....|....|
Stavropol_set_4_oligs      TAGAGTRTCYACICTMAAGG-----RCCYTCWAACACGCAYGTGCAYCA-----RAAGCAATGCCTGGARRCAG
Stavropol_U6               TAGAGTGTCCACGCTCAAGG-----ACCYTCAAACACGCATGTGCACCA-----GAAGCAATGCCTGGAGACAG
Stavropol_U7               TAGAGTGTCCACGCTCAAGG-----ACCTTCAAACACGCATGTGCACCA-----GAAGCAATGCCTGGAGACAG
Stavropol_U9               TAGAGTGTCCACGCTCAAGG-----ACCTTCAAACACGCACGTGCACCA-----GAAGCAATGCCTGGAGACAG
Stavropol_U10              TAGAGTGTCCACGCTCAAGG-----ACCTTCAAACACGCATGTGCACCA-----GAAGCAATGCCTGGAGACAG
Stavropol_U31              TAGAGTGTCCACACTCAAGG-----ACCTTCAAACACGCATGTGCATCA-----GAAGCAATGCCTGGAAACAG
Stavropol_U35              TAGAGTGTCCACGCTCAAGG-----ACCTTCAAACACGCATGTGCACCA-----GAAGCAATGCCTGGAGACAG
Stavropol_N96              TAGAGTGTCCACGCTCAAGG-----ACCTTCAAACACGCATGTGCACCA-----GAAGCAATGCCTGGAGACAG
Stavropol_N11              TAGAGTGTCCACGCTCAAGG-----GCCTTCAAACACGCATGTGCACCA-----GAAGCAATGCCTGGAAACAG
Stavropol_N12              TAGAGTGTCCACGCTCAAGG-----GCCTTCAAACACGCATGTGCACCA-----GAAGCAATGCCTGGAAACAG
Stavropol_N46              TAGAGTGTCCACGCTCAAGG-----------------------------------------------------
Stavropol_N48              TAGAGTGTCCACGCTCAAGG-----ACCTTCAAACACGcATGtGCaCCA-----GAAG----------------
Stavropol_N63              TAGAGTGTCCACGCTCAAGG-----GCCTTCTAACACGCATGTGCACCA-----GAAGCAATGCCTGGAAGCAG
Stavropol_N93              TAGAGTGTCCACGCTCAAGG-----ACCTTCAAACACGCATGTGCACCA-----GAAGCAATGCCTGGAGACAG
Stavropol_N95              TAGAGTGTCCACGCTCAAGG-----ACCTTCAAACACGCATGTGCACCA-----GAAGCAATGCCTGGAGACAG
Stavropol_N10              TAGAGTGTCCACGCTCAAGG-----GCCTTCAAACACGCACGTGCACCA-----GAAGCAATGCCTGGAAACAG
Stavropol_N4               TAGAGTGTCCACGCTCAAGG-----ACCTTCAAACACGCATGTGCACCA-----GAAGCAATGCCTGGAGACAG
Stavropol_N2               TAGAGTGTCCACGCTCAAGG-----GCCTTCAAACACGCACGTGCACCA-----GAAGCAATGCCTGGAAACAG
Stavropol_N3               TAGAGTGTCCACGCTCAAGG-----ACCTTCAAACACGCATGTGCACCA-----GAAGCAATGCCTGGAGACAG
```

```
Stavropol_N23        TAGAGTGTCCACGCTCAAGG-----GCCTTCAAACACGCATGTGCACCA-----GAAGCAATGCCTGGAAACAG
Stavropol_T961       TAGAGTGTCCACTCTCAAGG-----ACCTTCAAACACGCATGTGCACCA-----GAAGCAATGCCTGGAGACAG
Stavropol_T944       TAGAGTGTCCACTCTCAAGG----------------------------------------------------
Stavropol_Che321     TAGAGTGTCCACGCTCAAGG-----ACCTTCCAACACGCATGTGCACCA-----GAAGCAATGCCTGGAGACAG
Stavropol_Che325     TAGAGTGTCCACGCTCAAGG-----ACCTTCAAACACGCATGTGCACCA-----GAAGCAATGCCTGGAGACAG
Stavropol_Che320     TAGAGTGTCCACGCTCAAGG-----ACCTTCCAACACGCATGTGCACCA-----AAAGCAATGCCTGGAGACAG
Stavropol_Che324     TAGAGTGTCCACGCTCAAGG-----ACCTTCAAACACGCATGTGCACCA-----GAAGCARTGCCTGGAGACAG
Stavropol_1593       TAGAGTGTCCACGCTCAAGG-----GCCTTCTAACACGCATGTGCACCA-----GAAGCAATGCCTGGAAGCAG
Stavropol_1590       TAGAGTGTCTACGCTCAAGG-----GCCTTCTAACACGCATGTGCACCA-----GAAGCAATGCCTGGAAGCAG
Stavropol_K69        TAGAGTATCCACGCTCAAGG-----ACCTTCAAACACGCATGTTCACCA-----GAAGCAATGCCTGGAGACAG
Stavropol_k11        TAGAGTGTCCACGCTCAAGG-----ACCTTCTAACACGCATGTGCACCA-----AAAGCAATGCCTGGAGACAG
Stavropol_K62        TAGAGTATCCACGCTCAAGG-----ACCTTCAAACACGCATGTGCACCA-----GAAGCAATGCCTGGAGACAG
Stavropol_K59        TAGAGTGTCCACGCTCAAGG-----ACCCTCAAACACGCATGTGCACCA-----GAAGCAATGCCTGGAGACAG
Stavropol_k22        TAGAGTGTCCACGCTCAAGG-----ACCTTCAAACACGCAYGTGCACCA-----GAAGCAATGCCTGGAGACAG
Stavropol_k13        TAGAGTGTCCACGCTSAAGG-----ACCTTCAAACACGCATGTGCACCA-----GAAGCAATGCCTGGAGACAG
Stavropol_k14        TAGAGTGTCCACGCTCAAGG-----ACCTTCWAACACGCAYGTGCACCA-----GAAGCAATGCCTGGAGACAG
Stavropol_k10        TAGAGTGTCTACGCTAAAGG-----ACCTTCAAACACGCATGTGCACCA-----AAAGCAATGCCTGGAAACAG
Stavropol_K17        TAGAGTGTCCACGCTCAAGG-----ACCTTCAAACACGCAYGTGCACCA-----GAAGCAATGCCTGGAGACAG
Stavropol_k58        TAGAGTGTCCACGCTCAAGG-----ACCTTCAAACACGCACGTGCACCA-----GAAGCAATGCCTGGAGACAG
Stavropol_k60        TAGAGTGTCCACKCTCAAGG-----ACCATCAAACACGCATGTGCACCA-----GAAGCAATGCCTGGAGACAG
Stavropol_k61        TAGAGTGTCCACGCTCAAGG-----ACCTTCTAACACGCATGTGCACCA-----AAAGCAATGCCTGGAGACAG
Stavropol_k28        TAGAGTGTCCACGCTCAAGG-----ACCTTCAAACACGCATGTGCACCA-----GAAGCAATGCCTGGAGACAG
Stavropol_k63        TAGAGTGTCCACGCTCAAGG-----ACCTTCTAACACGCATGTGCACCA-----AAAGCAATGCCTGGAGACAG
Stavropol_k65        TAGAGTGTCCACTCTCAAGG-----ACCTTCAAACACGCATGTGCACCA-----GAAGCAATGCCTGGAGACAG
Stavropol_k66        TAGAGTGTCCACGCTCAAGG-----ACCTTCAAACACGCATGTGCACCA-----GAAGCAATGCCTGGAGACAG
Stavropol_k70        TAGAGTGTCCACGCTCAAGG-----ACCTTCAAACACGCATGTGCACCA-----GAAGCAATGCCTGGAGACAG
Stavropol_k73        TAGAGTGTCCACGCTCAAGG-----ACCTTCAAACACGCATGTGCACCA-----GAAGCAATGCCTGGAGACAG
Stavropol_k74        TAGAGTGTCCACGCTCAAGG-----ACCTTCTAACACGCATGTGCACCA-----GAAGCAATGCCTGGAGACAG
Stavropol_k75        TAGAGTGTCCACGCTCAAGG-----ACCTTCWAACACGCATGTGCACCA-----GAAGCAATGCCTGGAGACAG
Stavropol_K103       TAGAGTGTCCACGCTCAAGG-----ACCTTCAAACACGCATGTGCACCA-----GAAGCAATGCCTGGAGACAG
Stavropol_TB1040     TAGAGTGTCCACGCTCAAGG-----GCCTTCAAACACGCACGTGCACCA-----GAAGCAATGCCTGGAAACAG
```

**B.**

```
                           And-rtF1                And-rtPr1                  And-rtR1
                           2656                2675 2727                  2749 2760                2779
                           ....|....|....|....|/////....|....|....|....|...//////....|....|....|....|
Andropov_set_1_oligs       GAGTGATGGCTYGAAACAGT-----ACACCCATGTGCACCACGTTYTG-----TGGAGACCATTGAGAAGCGA
Andropov_A79               GAGTGATGGCTCGAAACAGT-----ACACCCATGTGCACCACGTTCTG-----TGGAGACCATTGAGAAGCGA
Andropov_A70               GAGTGATGGCTCGAAACAGT-----ACACCCATGTGCACCACGTTCTG-----TGGAGACCATTGAGAAGCGA
Andropov_1568              GAGTGATGGCTCGAAACAGT-----ACACCCATGTGCACCACGTTCTG-----TGGAGACCATTGAGAAGCGA
Andropov_A85               GAGTGATGGCTYGAAACAGT-----ACACCCATGTGCACCACGTTCTg-----TGGAGACCATTGAGAAGCGA
Andropov_A68               GAGTGATGGCTCGAAACAGT-----ACACCCATGTGCACCACGTTCTG-----TGGAGACCATTGAGAAGCGA
Andropov_A57               GAGTGATGGCTCGAAACAGT-----ACACCCATGTGCACCACGTTTTG-----TGGAGACCATTGAGAAGCGA
MN542373_Kharabali_virus   GAGTGATGGCTCGAAACAGT-----ACACCCATGTGCACCACGTTCTG-----TGGAGACCATTGAGAAGCGA
```

```
                           And-rtF2                    And-rtPr2                  And-rtR2
                           2364                 2384   2398                 2418  2452                 2472
                           ....|....|....|....|./////....|....|....|....|./////....|....|....|....|.
Andropov_set_2_oligs       ACTCAACTCCAGCACTCATCA-----ACTGGAGGACCARCACCCTGG-----WTTGGGTGACATGAGAACCCA
Andropov_A79               ACTCAACTCCAGCACTCATCA-----ACTGGAGGACCAGCACCCTGG-----ATTGGGTGACATGAGAACCCA
Andropov_A70               ACTCAACTCCAGCACTCATCA-----ACTGGAGGACCAACACCCTGG-----TTTGGGTGACATGAGAACCCA
Andropov_1568              ACTCAACTCCAGCACTCATCA-----ACTGGAGGACCAGCACCCTGG-----ATTGGGTGACATGAGAACCCA
MN542373_Kharabali_virus   ACTCAACTCCAGCACTCATCA-----ACTGGAGGACCAGCACCCTGG-----ATTGGGTGACATGAGAACCCA
Andropov_A85               --------------------------AcTGGAGGACcAGCAcCCTGG-----aTTGGGTGACATGAGAACCCA
Andropov_A68               --------------------------AcTGGAGGACCAACAcCCTGG-----tTTGGGTGACATGAGAACCCA
Andropov_A57               ----------------------------------------------------ATTGGGTGACATGAGAACCCA
```

## C.

```
                     Pd-rtF1                 Pd-rtPr1                    Pd-rtR1
                     2703             2721   2726                  2747  2780               2799
                     ....|....|....|...|/////....|....|....|....|..//////....|....|....|....|
Pedaselga_set_1_oligs  TCTCTGGGTGGCTACTCTC-----GCAGCTCGGAACGCTAAAGGCT-----CGATCCYAAGARGTSCYACC
Pedaselga_T16407       TCTCTGGGTGGCTACTCTC-----GCAGCTCGGAACGCTAAAGGCT-----CGATCCCAAGAGGTCCTACC
Pedaselga_T16417       TCTCTGGGTGGCTACTCTC-----GCAGCTCGGAACGCTAAAGGCT-----CGATCCCAAGAGGTCCTACC
Pedaselga_T16444       TCTCTGGGTGGCTACTCTC-----GCAGCTCGGAACGCTAAAGGCT-----CGATCCCAAGAGGTCCTACC
Pedaselga_T16446       TCTCTGGGTGGCTACTCTC-----GCAGCTCGGAACGCTAAAGGCT-----CGATCCCAAGAGGTCCTACC
Pedaselga_Ty545        TCTCTGGGTGGCTACTCTC-----GCAGCTCGGAACGCTAAAGGCA-----CGATCCCAAGAAGTCCTACC
Pedaselga_Ty518        TCTCTGGGTGGCTACTCTC-----GCAGCTCGGAACGCTAAAGGCT-----CGATCCTAAGAGGTCCTACC
Pedaselga_T16453       TCTCTGGGTGGCTACTCTC-----GCAGCTCGGAACGCTAAAGGCT-----CGATCCCAAGAGGTCCTACC
Pedaselga_T16454       TCTCTGGGTGGCTACTCTC-----GCAGCTCGGAACGCTAAAGGCT-----CGATCCCAAGAGGTCCTACC
Pedaselga_T27890       TCTCTGGGTGGCTACTCTC-----GCAGCTCGGAACGCTAAAGGCT-----CGATCCCAAGAGGTCCTACC
Pedaselga_k52          TCTCTGGGTGGCTACTCTC-----GCAGCTCGGAACGCTAAAGGCA-----CGATCCCAAGAAGTCCTACC
Pedaselga_Tu2_2Phd     TCTCTGGGTGGCTACTCTC-----GCAGCTCGGAACGCTAAAGGCA-----CGATCCCAAGAWGTSCYAYC
Pedaselga_Tu5_2Phd     TCTCTGGGTGGCTACTCTC-----GCAGCTCGGAACGCTAAAGGCT-----CGATCCCAAGAGGTSCYACC
Pedaselga_KT3763       TCTCTGGGTGGCTACTCTC-----GCAGCTCGGAACGCTAAAGGCT-----CGATCCCAAGAGGTCCTACC
Pedaselga_KT4295       TCTCTGGGTGGCTACTCTC-----GCAGCTCGGAACGCTAAAGGCT-----CGATCCCAAGAGGTCCTACC
Pedaselga_KT4374       TCTCTGGGTGGCTACTCTC-----GCAGCTCGGAACGCTAAAGGCT-----CGATCCCAAGAGGTCCTACC
Pedaselga_KT4437       TCTCTGGGTGGCTACTCTC-----GCAGCTCGGAACGCTAAAGGCT-----CGATCCCAAGAGGTCCTACC
Pedaselga_KT3724       TCTCTGGGTGGCTACTCTC-----GCAGCTCGGAACGCTAAAGGCT-----CGATCCCAAGAGGTCCTACC
MN542366_Onega_virus   TCTCTGGGTGGCTACTCTC-----GCAGCTCGGAACGCTAAAGGCA-----TGATCCCAAGAGGTCCTACC

                     Pd-rtF2                  Pd-rtPr2                        Pd-rtR2
                     2737              2756   2779                      2804  2841               2860
                     ....|....|....|....|/////....|....|....|....|....|./////....|....|....|....|
Pedaselga_set_2_oligs  CRCTAAAGGCWTCCTCAACC-----ACGATCCYAAGAWGTSCYACCACAGG-----GAGCACACTCATGTGCATGA
Pedaselga_T16407       CGCTAAAGGCTTCCTCAACC-----ACGATCCCAAGAGGTCCTACCACAGG-----GAGCACACTCATGTGCATGA
```

```
Pedaselga_T16444       CGCTAAAGGCTTCCTCAACC-----ACGATCCCAAGAGGTCCTACCACAGG-----GAGCACACTCATGTG-----
Pedaselga_T16446       CGCTAAAGGCTTCCTCAACC-----ACGATCCCAAGAGGTCCTACCACAGG-----GAGCACACTCATGTGCATGA
Pedaselga_T16453       CGCTAAAGGCTTCCTCAACC-----ACGATCCCAAGAGGTCCTACCACAGG-----GAGCACACTCATGTGCATGA
Pedaselga_T16454       CGCTAAAGGCTTCCTCAACC-----ACGATCCCAAGAGGTCCTACCACAGG-----GAGCACACTCATGTGC----
Pedaselga_K52          CGCTAAAGGCATCCTCAACC-----ACGATCCCAAGAAGTCCTACCACAGG-----GAGCACACTCATGTGCATGA
Pedaselga_T27890       CGCTAAAGGCTTCCTCAACC-----ACGATCCCAAGAGGTCCTACCACAGG-----GAGCACACTCATGTGCATGA
Pedaselga_Ty545        CGCTAAAGGCATCCTCAACC-----ACGATCCCAAGAAGTCCTACCACAGG-----GAGCACACTCATGTGCATGA
Pedaselga_Ty518        CGCTAAAGGCTTCCTCAACC-----ACGATCCTAAGAGGTCCTACCACAGG-----GAGCACACTCATGTGCATGA
Pedaselga_KT3763_1     CGCTAAAGGCTTCCTCAACC-----ACGATCCCAAGAGGTCCTACCACAGG-----GAGCACACTCATGTGCATGA
Pedaselga_KT3763_2     CGCTAAAGGCTTCCTCAACC-----ACGATCCCAAGAGGTSCTACCACAGG-----GAGCACACTCATGTGCATGA
Pedaselga_KT4295_2     CGCTAAAGGCTTCCTCAACC-----ACGATCCCAAGAGGTCCTACCACAGG-----GAGCACACTCATGTGCATGA
Pedaselga_KT4295_1     CGCTAAAGGCTTCCTCAACC-----ACGATCCCAAGAGGTCCTACCACAGG-----GAGCACACTCATGTGCATGA
Pedaselga_KT4437_2     CGCTAAAGGCTTCCTCAACC-----ACGATCCCAAGAGGTCCTACCACAGG-----GAGCACACTCATGTGCATGA
Pedaselga_KT4374_2     CGCTAAAGGCTTCCTCAACC-----ACGATCCCAAGAGGTCCTACCACAGG-----GAGCACACTCATGTGCATGA
Pedaselga_KT3724_2     CGCTAAAGGCTTCCTCAACC-----ACGATCCCAAGAGGTCCTACCACAGG-----GAGCACACTCATGTGCATGA
MN542366_Onega_virus   CGCTAAAGGCATCCTCAACC-----ATGATCCCAAGAGGTCCTACCACAGG-----GAGCACACTCATGTGCATGA
Pedaselga_T16417       CGCTAAAGGCTTCCTCAACC-----ACGATCCCAAGAGGTCCTACCACAGG-------------------------
Pedaselga_KT3724_1     CGCTAAAGGCTTCCTCAACC-----ACGATCCCAAGAGGTCCTACCACAGG-------------------------
Pedaselga_Tu5_2        CGCTAAAGGCTTCCTCAACC-----ACGATCCCAAGAGGTSCYACCACAGG-------------------------
Pedaselga_Tu2_2        CGCTAAAGGCATCCTCAACC-----ACGATCCCAAGAWGTSCYACCACAGG-------------------------
Pedaselga_kt3856       CRCTAAAG--------------------------------------------------------------------
```

**D.**

```
                     Kz-rtF1                   Kz-rtPr1                         Kz-rtR1
                     2726               2745 2816                      2842 2847               2866
                     ....|....|....|....|/////....|....|....|....|....|../////....|....|....|....|
Kizhi_set_1_oligs    ATCAGCCACGTACGATCAGA-----TCACAGAGCAACCCACATYCATGAGCT-----CAGGAGTGTTTGGAGGGACT
Kizhi_T16445         ATCAGCCACGTACGATCAGA-----TCACAGAGCAACCCACATTCATGAGCT-----CAGGAGTGTTTGGAGGGACT
Kizhi_T16442         ATCAGCCACGTACGATCAGA-----TCACAGAGCAACCCACATTCATGAGCT-----CAGGAGTGTTTGGAGGGACT
Kizhi_T16398         ATCAGCCACGTACGATCAGA-----TCACAGAGCAACCCACATTCATGAGCT-----CAGGAGTGTTTGGAGGGACT
Kizhi_T16404         ATCAGCCACGTACGATCAGA-----TCACAGAGCAACCCACATTCATGAGCT-----CAGGAGTGTTTGGAGGGACT
MN542368_Sara_virus  ATCAGCCACGTACGATCAGA-----TCACAGAGCAACCCACATTCATGAGCT-----CAGGAGTGTTTGGAGGGACT
Kizhi_T16346         ATCAGCCACGTACGATCAGA---------------------------------------------------------

                     Kz-rtF2                   Kz-rtPr2                    Kz-rtR2
                     2598               2617 2681                  2703 2715               2734
                     ....|....|....|....|/////....|....|....|....|.../////....|....|....|....|
Kizhi_set_2_oligs    TACTGTGCTGACTCTGCCAY-----TGCCATTGGCAACTACGACCTCG-----ACACTGAAAGCATCAGCCAC
Kizhi_T16442         TACTGTGCTGACTCTGCCAT-----TGCCATTGGCAACTACGACCTCG-----ACACTGAAAGCATCAGCCAC
Kizhy_T16445         TACTGTGCTGACTCTGCCAT-----TGCCATTGGCAACTACGACCTCG-----ACACTGAAAGCATCAGCCAC
Kizhi_T16404         TACTGTGCTGACTCTGCCAT-----TGCCATTGGCAACTACGACCTCG-----ACACTGAAAGCATCAGCCAC
Kizhi_T16346         TACTGTGCTGACTCTGCCAC-----TGCCATTGGCAACTACGACCTCG-----ACACTGAAAGCATCAGCCAC
Kizhi_T16398         TACTGTGCTGACTCTGCCAT-----TGCCATTGGCAACTACGACCTCG-----ACACTGAAAGCATCAGCCAC
```

```
MN542368_Sara_virus   TACTGTGCTGACTCTGCCAT-----TGCCATTGGCAACTACGACCTCG-----ACACTGAAAGCATCAGCCAC
```

**E.**

```
                         Gm-rtF2                  Gm-rtPr2                      Gm-rtR2
                         2573                2592 2613                     2637 2680                2699
                         ....|....|....|....|/////....|....|....|....|....|/////....|....|....|....|
Gomselga_set_2_oligs     CCACCCTHAAGGCCACATCR-----TGTRCACTCYGARAACAAGGGAMGA-----ACRAGRGCAGGAAAGAGRCT
Gomselga_T20618-21       CCACCCTCAAGGCCACATCA-----TGTGCACTCTGAAAACAAGGGACGA-----ACAAGGGCAGGAAAGAGACT
Gomselga_T28815          CCACCCTTAAGGCCACATCG-----TGTGCACTCCGAGAACAAGGGAAGA-----ACGAGAGCAGGAAAGAGACT
Gomselga_T28132          CCACCCTAAAGGCCACATCG-----TGTACACTCTGAGAACAAGGGAAGA-----ACGAGGGCAGGAAAGAGACT
Gomselga_T27359          CCACCCTTAAGGCCACATCA-----TGTGCACTCTGAGAACAAGGGACGA-----ACAAGAGCAGGAAAGAGGCT
Gomselga_T20501-504      CCACCCTTAAGGCCACATCA-----TGTGCACTCTGAGAACAAGGGACGA-----ACAAGAGCAGGAAAGAGGCT
Gomselga_T27077          CCACCCTAAAGGCCACATCG-----TGTGCACTCTGAGAACAAGGGAAGA-----ACGAGGGCAGGAAAGAGACT
Gomselga_T28607          CCACCCTTAAGGCCACATCA-----TGTGCACTCTGAGAACAAGGGACGA-----ACAAGGGCAGGAAAGAGACT
Gomselga_T27345          CCACCCTTAAGGCCACATCA-----TGTGCACTCTGAGAACAAGGGACGA-----ACAAGAGCAGGAAAGAGGCT
Gomselga_T22645          CCACCCTTAAGGCCACATCA-----TGTGCACTCTGAGAACAAGGGACGA-----ACAAGAGCAGGAAAGAGACT
Gomselga_T22634          CCACCCTTAAGGCCACATCA-----TGTGCACTCTGAGAACAAGGGACGA-----ACAAGAGCAGGAAAGAGACT
Gomselga_T22248          CCACCCTTAAGGCCACATCG-----TGTGCACTCTGAGAATAAGGGACGA-----ACAAGAGCAGGAAAGAGACT
Gomselga_T16420          CCACCCTTAAGGCCACATCA-----TGTGCACTCTGAGAACAAGGGACGA-----ACAAGAGCAGGAAAGAGGCT

                         Gm-rtF3                  Gm-rtPr3                      Gm-rtR3
                         2615                2634 2667                     2690 2705                2724
                         ....|....|....|....|/////....|....|....|....|..../////....|....|....|....|
Gomselga_set_3_oligs     TRCACTCYGARAACAAGGGA-----AGTTGCMCCRYTGACRAGRGCAGG-----TAGAGGCATTYCAGGAGAGC
Gomselga_T20618-21       TGCACTCTGAAAACAAGGGA-----AGTTGCCCCATTGACAAGGGCAGG-----TAGAGGCATTCCAGGAGAGC
Gomselga_T16420          TGCACTCTGAGAACAAGGGA-----AGTTGCACCGTTGACAAGAGCAGG-----TAGAGGCATTCCAGGAGAGC
Gomselga_T22645          TGCACTCTGAGAACAAGGGA-----AGTTGCACCGTTGACAAGAGCAGG-----TAGAGGCATTCCAGGAGAGC
Gomselga_T22248          TGCACTCTGAGAATAAGGGA-----AGTTGCTCCATTGACAAGAGCAGG-----TAGAGGCATTCCAGGAGAGC
Gomselga_T22634          TGCACTCTGAGAACAAGGGA-----AGTTGCACCGTTGACAAGAGCAGG-----TAGAGGCATTCCAGGAGAGC
Gomselga_T27345          TGCACTCTGAGAACAAGGGA-----AGTTGCACCGTTGACAAGAGCAGG-----TAGAGGCATTCCAGGAGAGC
Gomselga_T27077          TGCACTCTGAGAACAAGGGA-----AGTTGCACCGCTGACGAGGGCAGG-----TAGAGGCATTTCAGGAGAGC
Gomselga_T28607          TGCACTCTGAGAACAAGGGA-----AGTTGCACCGCTGACAAGGGCAGG-----TAGAGGCATTCCAGGAGAGC
Gomselga_T27359          TGCACTCTGAGAACAAGGGA-----AGTTGCACCGTTGACAAGAGCAGG-----TAGAGGCATTCCAGGAGAGC
Gomselga_T20501-504      TGCACTCTGAGAACAAGGGA-----AGTTGCACCGTTGACAAGAGCAGG-----TAGAGGCATTCCAGGAGAGC
Gomselga_T28132          TACACTCTGAGAACAAGGGA-----AGTTGCACCGCTGACGAGGGCAGG-----TAGAGGCATTTCAGGAGAGC
Gomselga_T28815          TGCACTCCGAGAACAAGGGA-----AGTTGCACCGCTGACGAGAGCAGG-----TAGAGGCATTTCAGGAGAGC

                         Gm-rtF5                  Gm-rtPr5                      Gm-rtR5
                         2392                2411 2412                     2437 2546                2565
                         ....|....|....|....|/////....|....|....|....|....|./////....|....|....|....|
Gomselga_set_5_oligs     RGGTAYCTAGGGAARCAGGA-----YCCTSTRAAGCCYACYATGCATGAGT-----TCTGCCACCTCACYCTTGAG
```

```
Gomselga_T28815        AGGTATCTAGGGAAGCAGGA-----CCCTCTGAAGCCTACCATGCATGAGT-----TCTGCCACCTCACCCTTGAG
Gomselga_T20618-21     AGGTATCTAGGGAAGCAGGA-----TCCTGTGAAGCCCACTATGCATGAGT-----TCTGCCACCTCACTCTTGAG
Gomselga_T22248        AGGTATCTAGGGAAGCAAGA-----CCCTGTAAAGCCTACCATGCATGAGT-----TCTGCCACCTCACTCTTGAG
Gomselga_T22634        AGGTATCTAGGGAAACAGGA-----CCCTGTGAAGCCTACCATGCATGAGT-----TCTGCCACCTCACTCTTGAG
Gomselga_T22645        AGGTATCTAGGGAAACAGGA-----CCCTGTGAAGCCTACCATGCATGAGT-----TCTGCCACCTCACTCTTGAG
Gomselga_T27077        AGGTACCTAGGGAAGCAGGA-----CCCTGTGAAGCCTACCATGCATGAGT-----TCTGCCACCTCACTCTTGAG
Gomselga_T28607        AGGTATCTAGGGAAACAGGA-----CCCTGTAAAGCCTACCATGCATGAGT-----TCTGCCACCTCACTCTTGAG
Gomselga_T27359        AGGTATCTAGGGAAGCAGGA-----CCCTGTGAAGCCTACCATGCATGAGT-----TCTGCCACCTCACTCTTGAG
Gomselga_T20501-504    AGGTATCTAGGGAAGCAGGA-----CCCTGTGAAGCCTACCATGCATGAGT-----TCTGCCACCTCACTCTTGAG
Gomselga_T28132        AGGTACCTAGGGAAGCAGGA-----CCCTGTGAAGCCTACCATGCATGAGT-----TCTGCCACCTCACTCTTGAG
Gomselga_T27345        AGGTATCTAGGGAAGCAGGA-----CCCTGTGAAGCCTACCATGCATGAGT-----TCTGCCACCTCACTCTTGAG
Gomselga_T16420        AGGTATCTAGGGAAGCAGGA-----CCCTGTGAAGCCTACCATGCATGAGT-----TCTGCCACCTCACTCTTGAG
Gomselga_T16420_clone  GGGTATCTAGGGAAGCAGGA-----CCCTGTGAAGCCTACCATGCATGAGT-----TCTGCCACCTCACTCTTGAG
```

**Figure S1.** Fragments of L segment alignment. Sequence alignment was done using the ClustalW algorithm as implemented in the Bioedit package. Forward primers (-rtF-) and reverse primers (-rtR-) are designed to amplify a conservative part of the L segment of the genome. The hydrolysis probes (-rtPr-) are designed to bind to the complementary region within the amplified sequence. Fragment of phenuivirus L segment alignment: (A) nt positions, according to the *Dermacentor reticulatus uukuvirus* isolate CT4 genome, NCBI accession number: ON684362. The primers and probes binding sites for Stavropol detecting assays 1, 2, 4 are indicated above. (B) nt positions, according to the L segment of the *Kharabali tick phlebovirus;* NCBI accession number: MN542373. The primers and probes binding sites for Andropov detecting assays 1 and 2 are indicated above. (C) nt positions, according to the L segment of the *Onega tick phlebovirus;* NCBI accession number: ON408160. The primers and probes binding sites for Pedaselga detecting assays 1 and 2 are indicated above. (D) nt positions, according to the L segment of the *Sara tick phlebovirus;* NCBI accession number: MN542368. The primers and probes binding sites for Pedaselga detecting assays 1 and 2 are indicated above. (E) nt positions, according to the L segment of the *Mukawa virus;* NCBI accession number: NC_043510. The primers and probes binding sites for Pedaselga detecting assays 2, 3 and 5 are indicated above.

**Table S1.** Threshold cycle values for developed test systems.

| **Test system** | Ct* Stavropol MT380797 | Ct* Stavropol MT380783 | Ct* Andropov MT380747 | Ct* Andropov MT380748 | Ct* Pedaselga MT380766 | Ct* Pedaselga MT380768 | Ct* Kizhi MT380761 | Ct* Kizhi MT380762 | Ct* Gomselga ON920441 | Ct* Gomselga ON920443 |
|---|---|---|---|---|---|---|---|---|---|---|
| St-rt1 | 27,4 ± 1,4 | 31,3 ± 0,8 | - | - | - | - | - | - | - | - |
| St-rt2 | N/A | N/A | - | - | - | - | - | - | - | - |
| St-rt4 | 22,5 ± 0,7 | 18,6 ± 0,1 | - | - | - | - | - | - | - | - |
| And-rt1 | - | - | 32,4 ± 0,7 | 33,2 ± 1,5 | - | - | - | - | - | - |
| And-rt2 | - | - | 23,0 ± 0,6 | 25,3 ± 1,2 | - | - | - | - | -- | - |
| Pd-rt1 | - | - | - | - | 27,8 ± 0,7 | 26,3 ± 0,7 | - | - | - | - |
| Pd-rt2 | - | - | - | - | 30,5 ± 0,5 | 29,5 ± 1,1 | - | - | - | - |
| Kz-rt1 | - | - | - | - | - | - | 7,9 ± 0,6 | 9,9 ± 1,3 | - | - |
| Kz-rt2 | - | - | - | - | - | - | 5,9 ± 0,8 | 6,9 ± 0,5 | - | - |
| Gom-rt2 | - | - | - | - | - | - | - | - | N/A | 26,8 ± 1,6 |
| Gom-rt3 | - | - | - | - | - | - | - | - | 21,1 ± 1,2 | 18,1 ± 0,5 |
| Gom-rt5 | - | - | - | - | - | - | - | - | 26,4 ± 1,4 | 20,4 ± 0,9 |

* Average Ct of two RT-PCR runs.

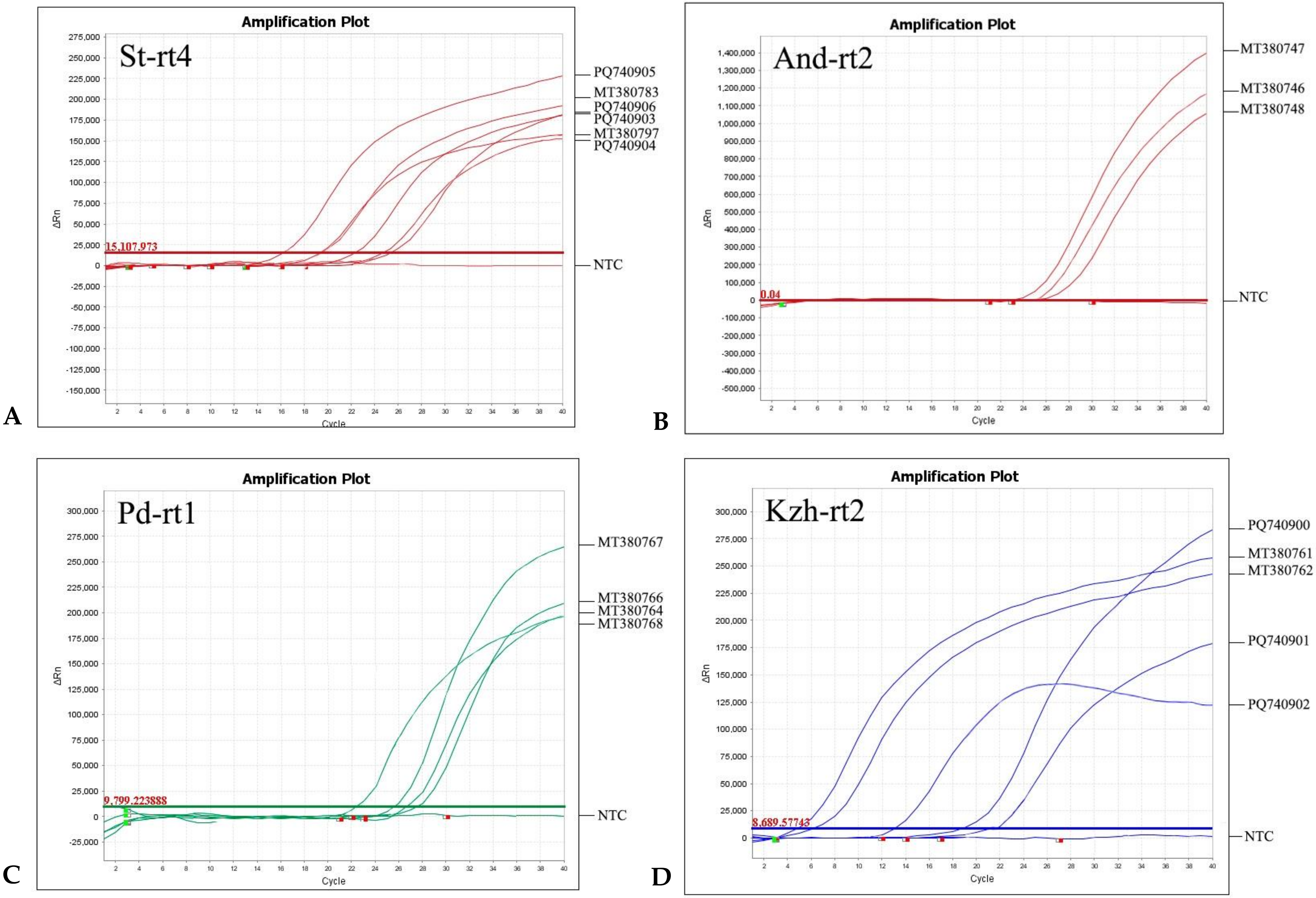
A
Amplification Plot
St-rt4
15,107.973
PQ740905
MT380783
PQ740906
PQ740903
MT380797
PQ740904
NTC
ΔRn
Cycle
B
Amplification Plot
And-rt2
0.04
MT380747
MT380746
MT380748
NTC
ΔRn
Cycle
C
Amplification Plot
Pd-rt1
9,799.223888
MT380767
MT380766
MT380764
MT380768
NTC
ΔRn
Cycle
D
Amplification Plot
Kzh-rt2
8,689.57743
PQ740900
MT380761
MT380762
PQ740901
PQ740902
NTC
ΔRn
Cycle

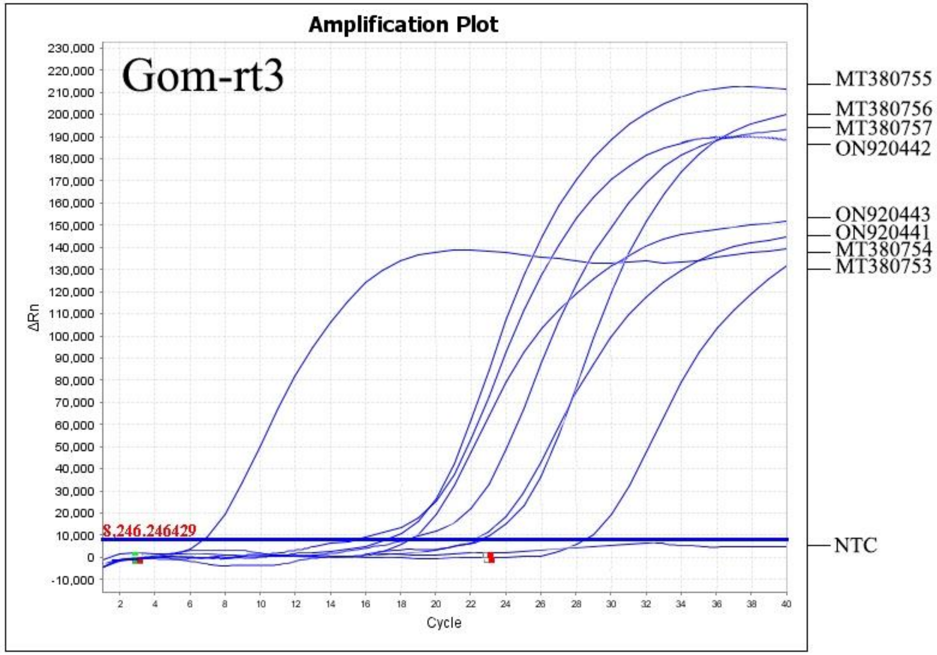


E

**Figure S2.** Fluorescence curves on the optical channels of the QuantStudio5 amplifier. The graphs show the curves for each sample with the lowest Ct among the three runs. Fluorescence was detected in samples containing the genetic material of: (A) Stavropol virus – 6 samples, FAM channel; (B) Andropov virus – 3 samples, ROX channel; (C) Pedaselga virus – 4 samples, FAM channel; (D) Kizhi virus – 5 samples, FAM channel; (E) Gomselga virus – 8 samples, Cy5 channel.

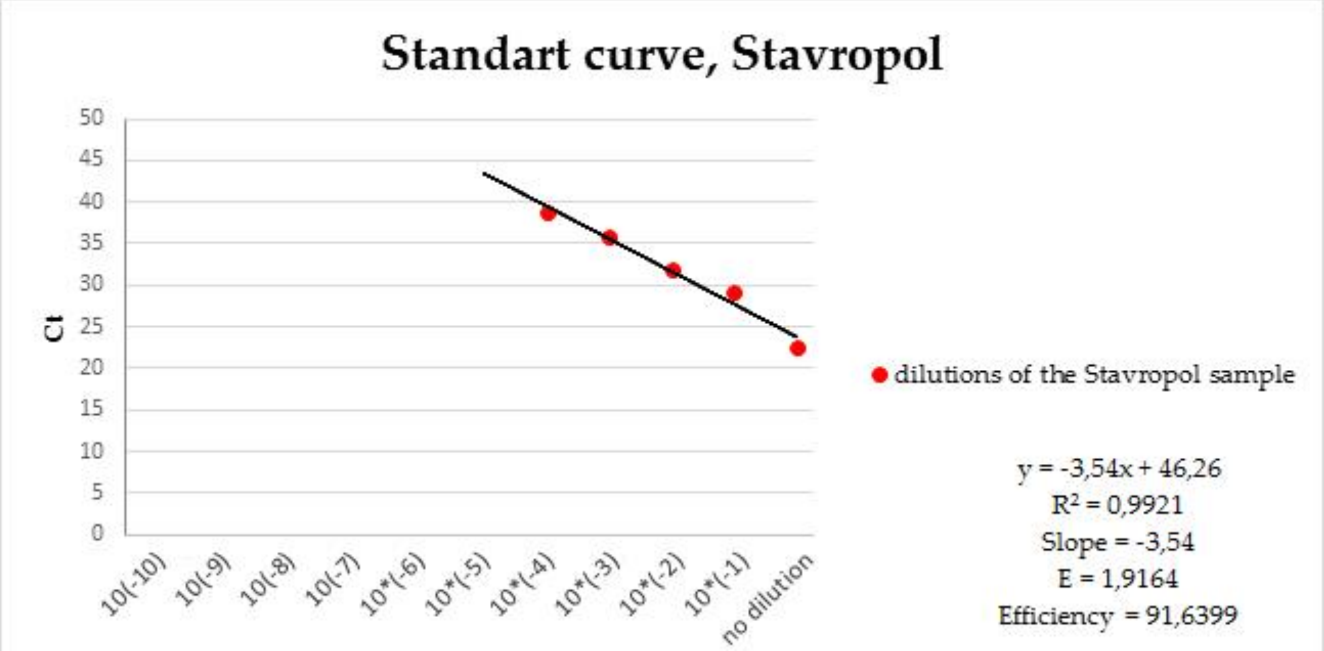


**Figure S3.** The linear dependence between the dilution degree and the threshold cycle of the control sample contained Stavropol virus. The Y-axis shows the number of cycles. The X-axis shows the degree of sample dilution.

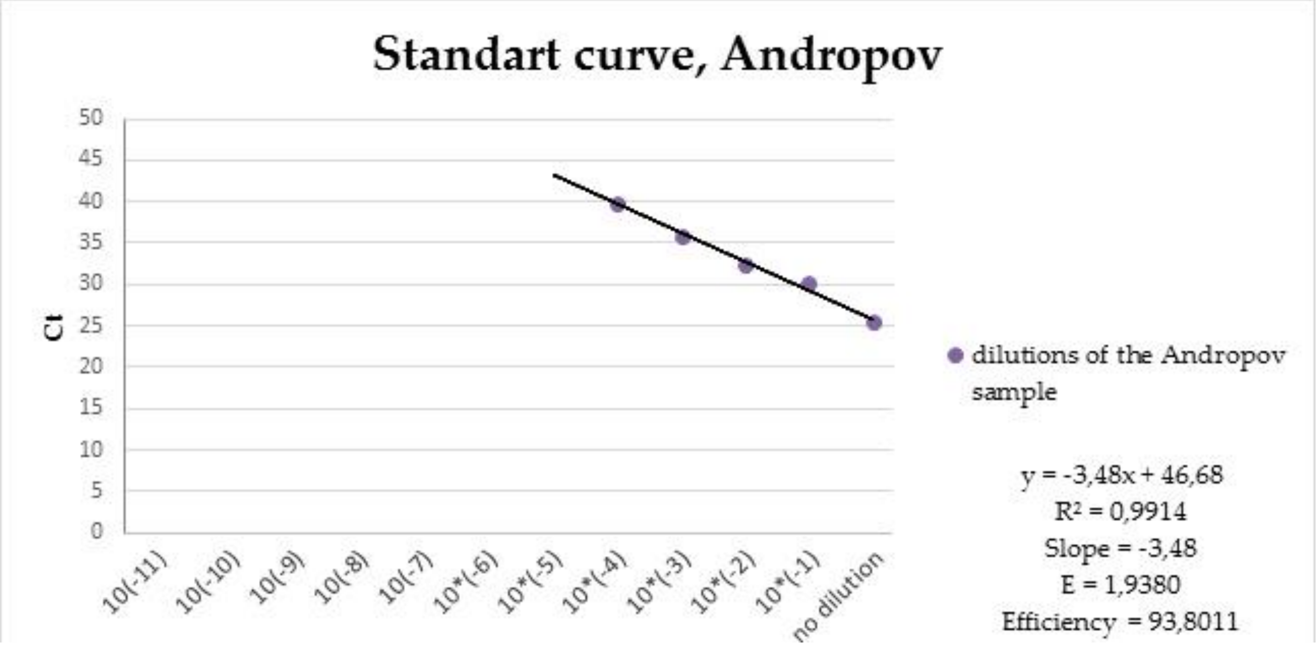


**Figure S4.** The linear dependence between the dilution degree and the threshold cycle of the control sample contained Andropov virus. The Y-axis shows the number of cycles. The X-axis shows the degree of sample dilution.

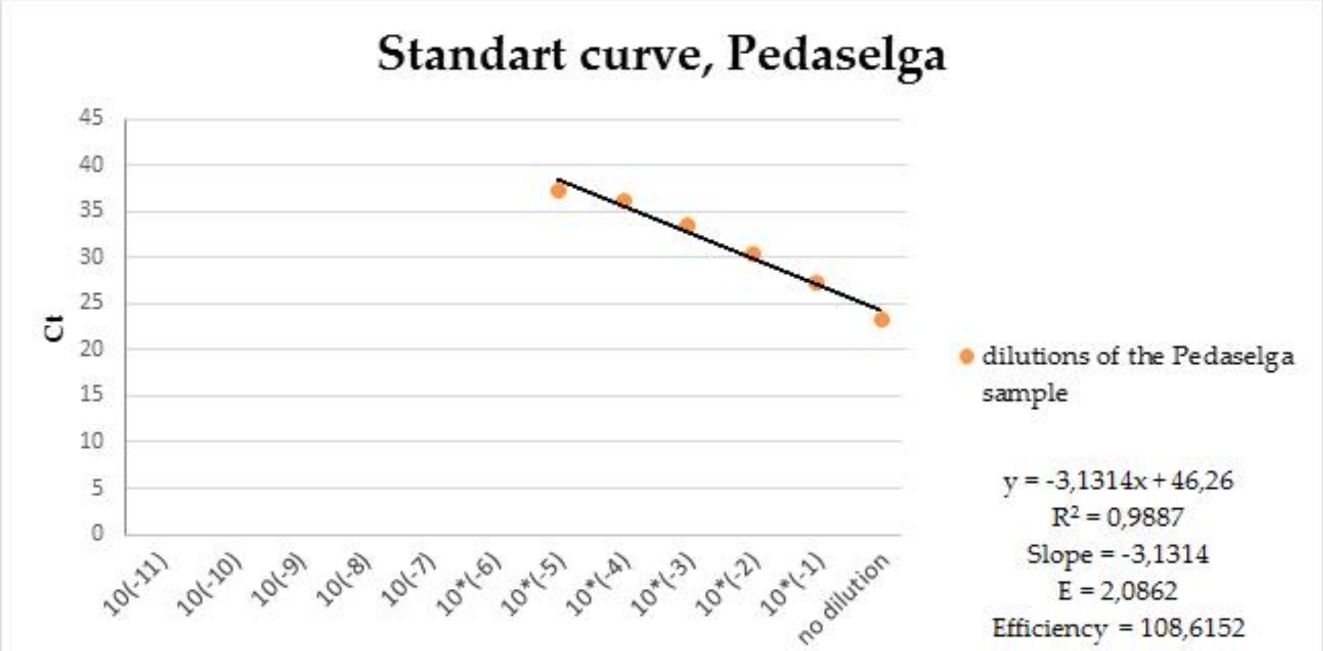


**Figure S5.** The linear dependence between the dilution degree and the threshold cycle of the control sample contained Pedaselga virus. The Y-axis shows the number of cycles. The X-axis shows the degree of sample dilution.

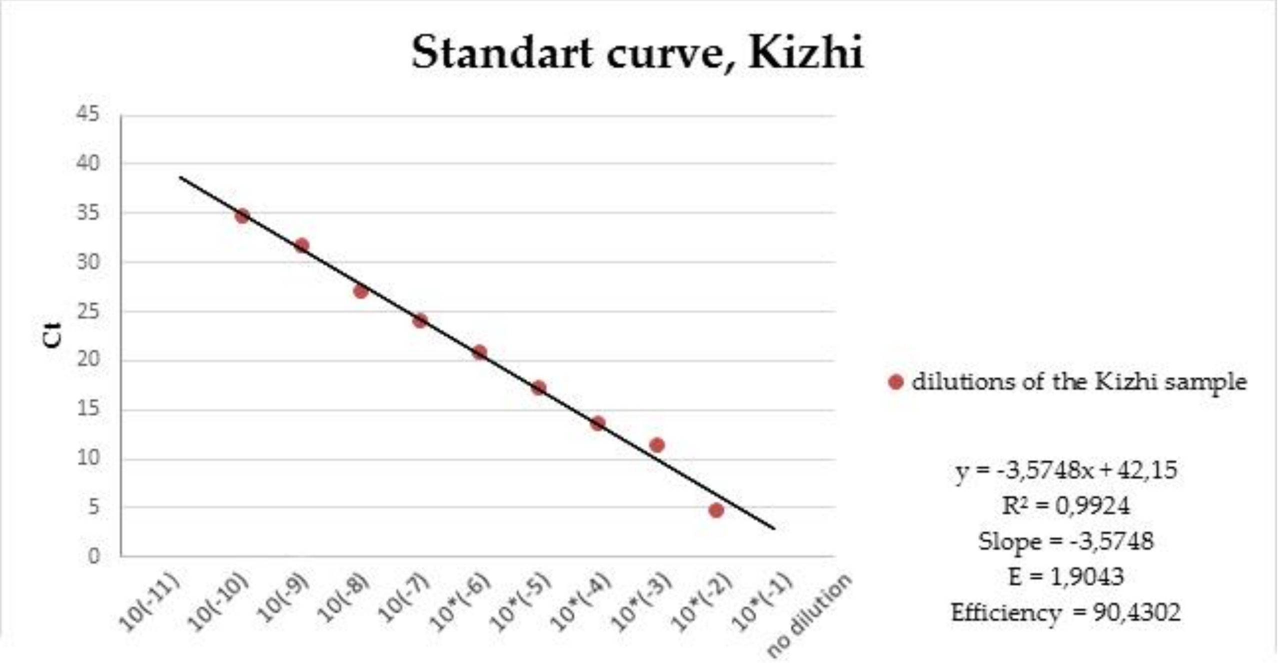


**Figure S6.** The linear dependence between the dilution degree and the threshold cycle of the control sample contained Kizhi virus. The Y-axis shows the number of cycles. The X-axis shows the degree of sample dilution.

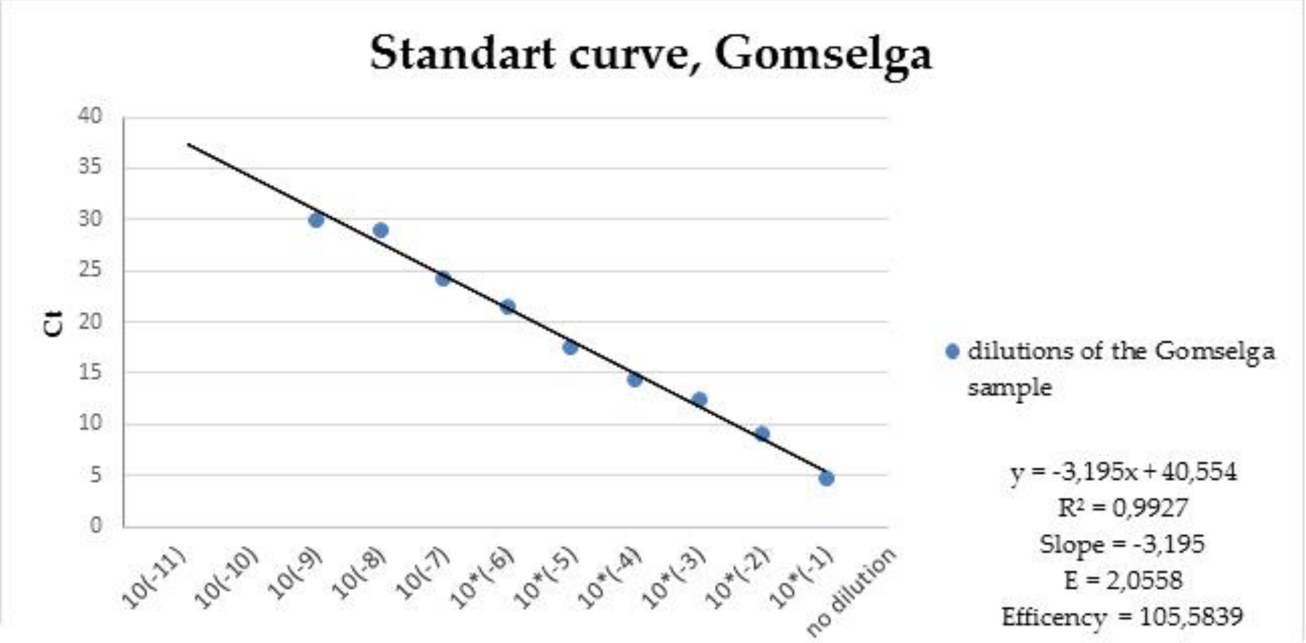


**Figure S7.** The linear dependence between the dilution degree and the threshold cycle of the control sample contained Gomselga virus. The Y-axis shows the number of cycles. The X-axis shows the degree of sample dilution.

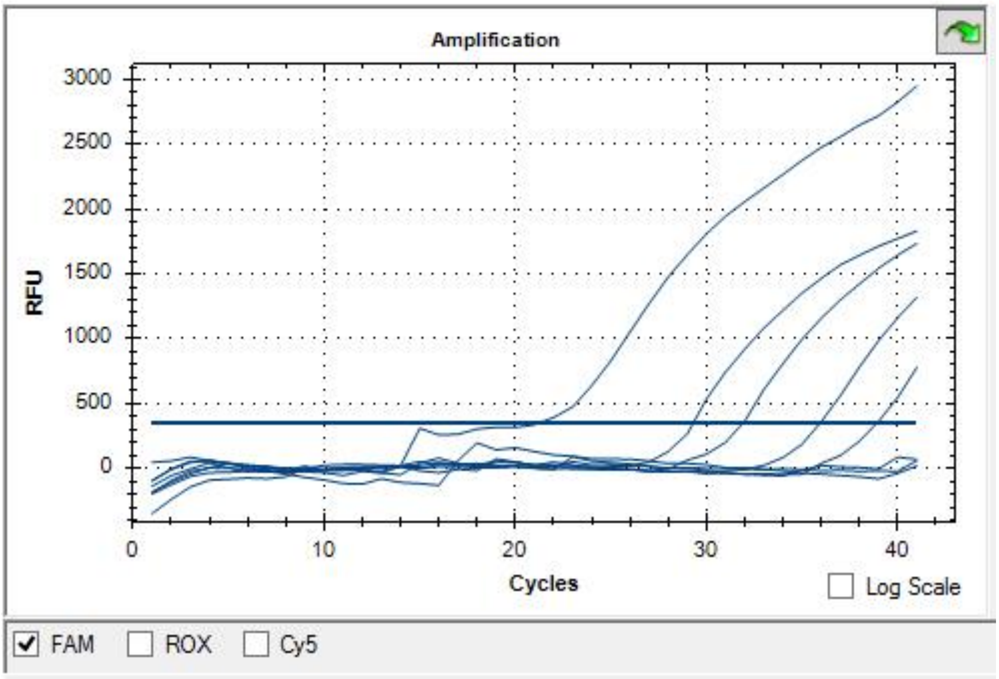


<u>Stavropol virus,</u>

<u>set St-rt4</u>

**Figure S8.** Amplification curve for the dilutions of the Stavropol virus. The sample was tested at the following dilutions: undiluted, $10^{-1}$, $10^{-2}$, $10^{-3}$, $10^{-4}$, $10^{-5}$, and $10^{-6}$. The order of the dilutions corresponds to the values provided in Table 4.

Andropov virus,

set And-rt2

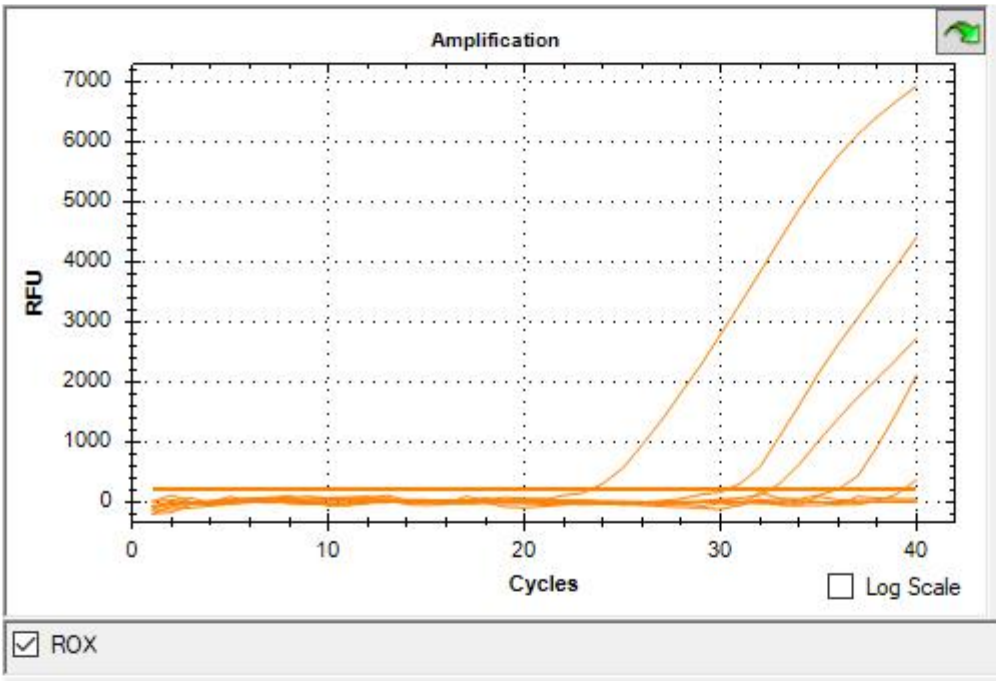


**Figure S9.** Amplification curve for the dilutions of the Andropov virus. The sample was tested at the following dilutions: undiluted, $10^{-1}$, $10^{-2}$, $10^{-3}$, $10^{-4}$, $10^{-5}$, and $10^{-6}$. The order of the dilutions corresponds to the values provided in Table 4.

Pedaselga virus,

set Pd-rt1

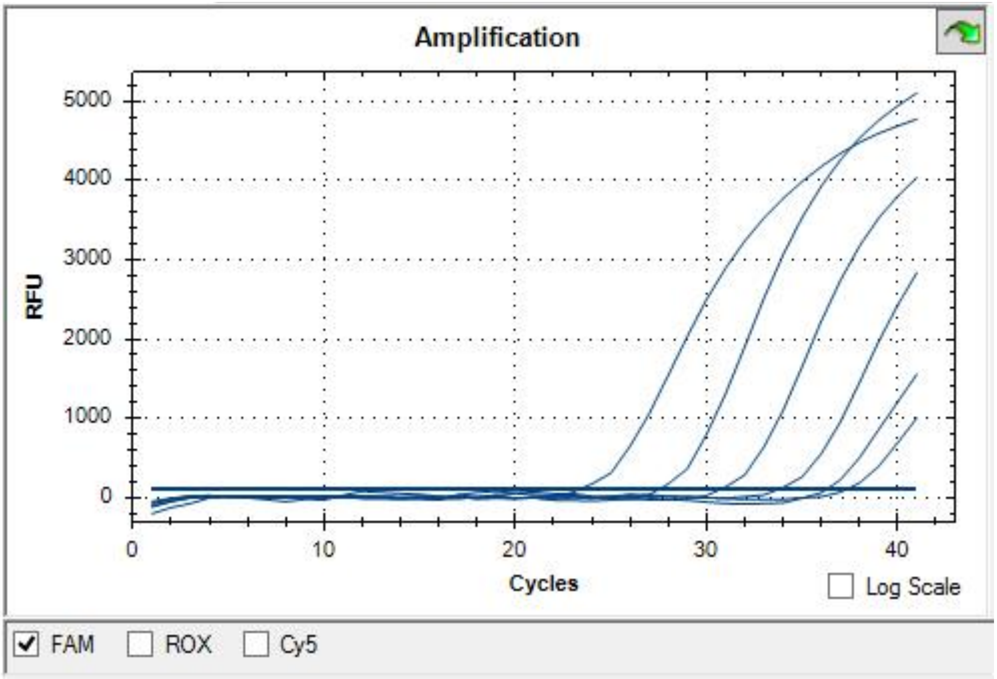


**Figure S10.** Amplification curve for the dilutions of the Pedaselga virus. The sample was tested at the following dilutions: undiluted, $10^{-1}$, $10^{-2}$, $10^{-3}$, $10^{-4}$, $10^{-5}$, and $10^{-6}$. The order of the dilutions corresponds to the values provided in Table 4.

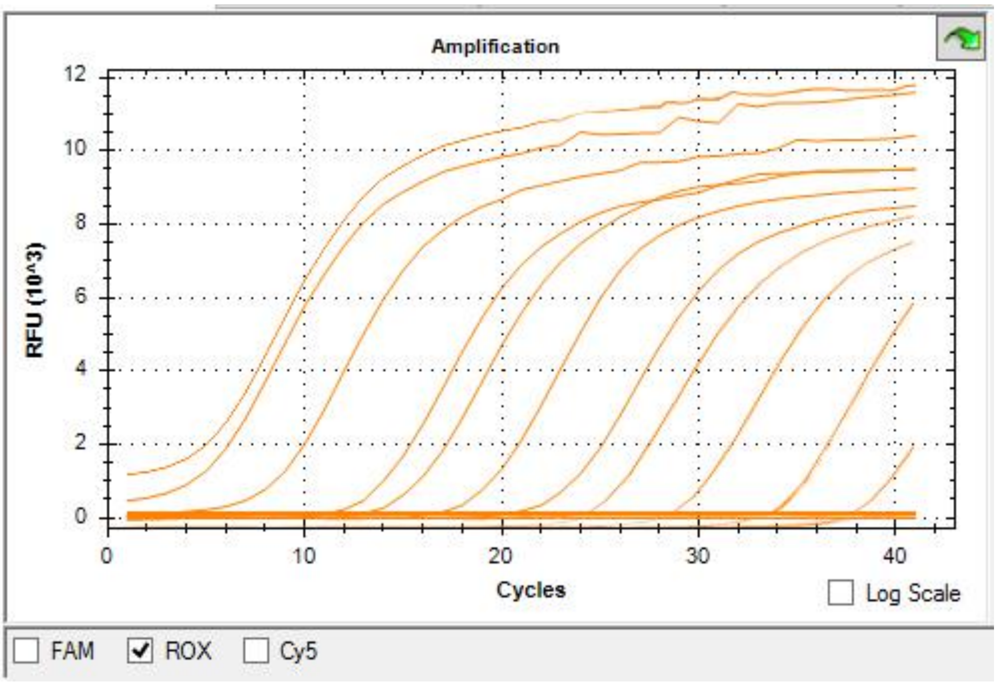


Kizhi virus,

set Kz-rt2

**Figure S11.** Amplification curve for the dilutions of the Kizhi virus. The sample was tested at the following dilutions: undiluted, $10^{-1}$, $10^{-2}$, $10^{-3}$, $10^{-4}$, $10^{-5}$ $10^{-6}$, $10^{-7}$, $10^{-8}$, $10^{-9}$, $10^{-10}$ and $10^{-11}$. The order of the dilutions corresponds to the values provided in Table 4.

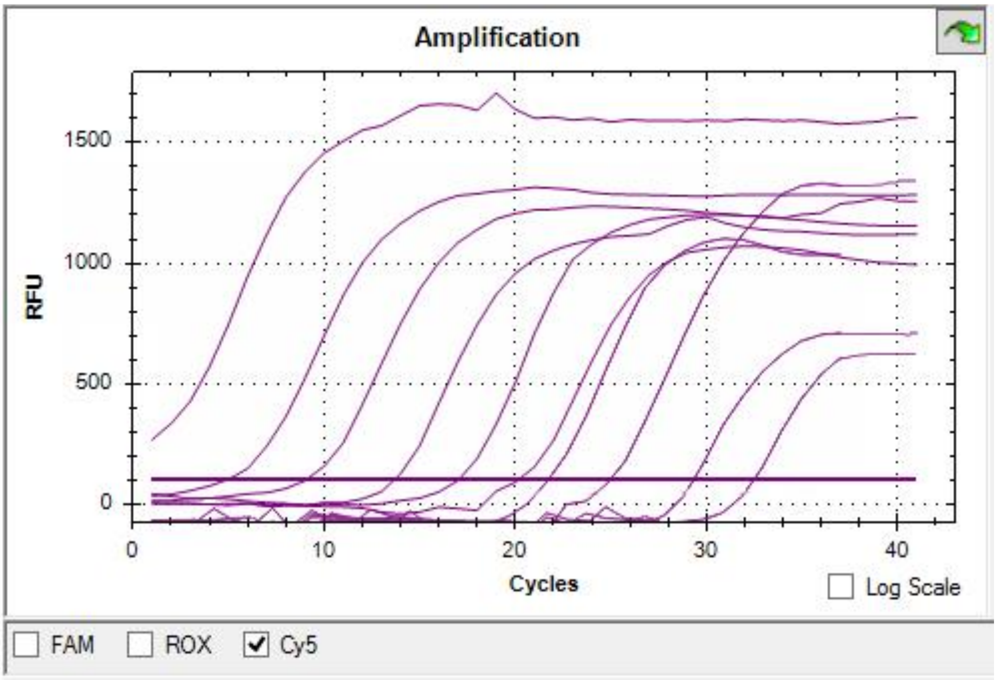


Gomselga virus,

set Gom-rt3

**Figure S12.** Amplification curve for the dilutions of the Gomselga virus. The sample was tested at the following dilutions: undiluted, $10^{-1}$, $10^{-2}$, $10^{-3}$, $10^{-4}$, $10^{-5}$ $10^{-6}$, $10^{-7}$, $10^{-8}$, $10^{-9}$, $10^{-10}$ and $10^{-11}$. The order of the dilutions corresponds to the values provided in Table 4.
